# Novel Crown Ether-Functionalized Fusidic Acid Butyl Ester: Synthesis, Biological Evaluation, In Silico ADMET, and Molecular Docking Studies

**DOI:** 10.3390/molecules30092033

**Published:** 2025-05-02

**Authors:** Hira Sultan, Nuzhat Arshad, Mehreen Lateef

**Affiliations:** 1 Department of Chemistry, NED University of Engineering and Technology, Karachi 75270, Pakistan; hirasultan@cloud.neduet.edu.pk; 2 Multidisciplinary Lab, Bahria University of Karachi, Karachi 75270, Pakistan; mehreen.lateef80@gmail.com

**Keywords:** Fusidic acid butyl ester-linked crown ethers, α-glucosidase inhibition, antioxidant activity, molecular docking, ADMET studies

## Abstract

Crown ethers have gained importance in the field of medicine because of their resemblance to natural ionophores like valinomycin. With the goal of developing new pharmacologically important crown ethers, a novel series of crown ethers linked with Fusidic acid butyl ester **10a**–**d** were synthesized and characterized by means of their ^1^H NMR, ^13^C NMR DEPT-135, FT-IR, and mass spectrometry. In vitro antioxidant and α-glucosidase inhibition activities of all crown ethers along with the precursor Fusidic acid butyl ester were examined and compared to the standard butylated hydroxyanisole and acarbose, respectively. Compounds (FABE-16-crown-4) **10b** and (FABE-19-crown-5) **10c** showed high antioxidant potential with the IC_50_ = 22.5 ± 0.2 μM and 32.1 ± 0.3 μM, respectively, when compared to the standard BHA (IC_50_ = 44.2 ± 0.34 μM). To understand the binding mode of the compounds, molecular docking investigations were performed using human antioxidant protein, peroxiredoxin 5. Molecular docking studies revealed higher docking scores (−6.5 and −6.7 kcal/mol) for the highly active compounds **10c** and **10b**, respectively, than standard BHA (−5.3 kcal/mol). Synthesized crown ethers exhibited moderate α-glucosidase inhibition with (IC_50_ = 23.5 ± 0.2 to 76.5 ± 0.1 μM) when compared to acarbose as standard (IC_50_ = 5.2 ± 0.8 μM). The in silico ADMET predictions indicated that the prepared compounds obeyed (bRO5) and Veber’s rule for the acceptance as orally administered drugs and indicated that all the prepared crown ethers exhibited calculated values of drug likeness parameters in acceptable ranges that showed good potential of these molecules for further drug development investigations.

## 1. Introduction

Crown ethers have established their applications across a wide range of scientific fields, including mass spectrometry [1], catalysis [2], chromatography [3], dyes, chemo sensors, materials sciences, electrochemical switches [4], environmental restoration, and, recently, nanoscience. Most significantly, they have gained importance in the field of medicine, owing to their capability of making non-covalent interactions with biological cations like Ca^+2^, K^+^, Na^+^ and biopolymers, giving them a strong resemblance to natural ionophores like valinomycin and gramicidin [5]. Nevertheless, exploring biologically active synthetic molecules that are structurally, functionally, and mechanistically similar to natural analogs remains a fundamental research interest [6]. Hence, various structurally modified crown ethers have been developed and mechanistically studied for therapeutic purposes [7], such as antioxidant [8], anti-cancer [9], anti-inflammatory [10], antidiabetic [11], and antibiotic [12,13] activities.

**Figure 1:**
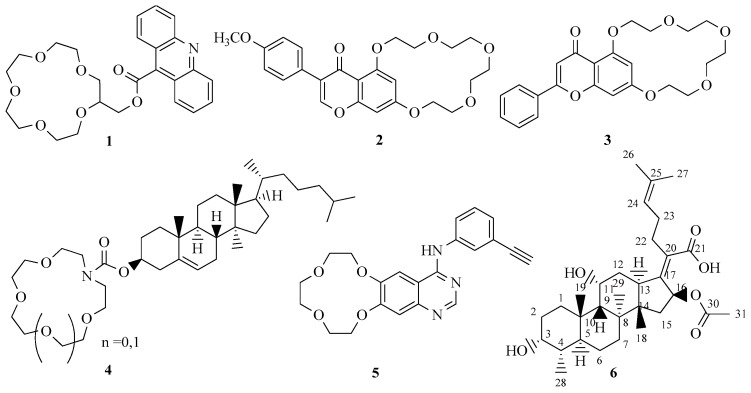
Natural product-linked crown ethers. Acridine crown ethers (anti-cancer) **1** [18], Biochanin A-linked crown ether (anti-cancer) **2** [19], Chrysin-linked crown ether (antidiabetic) **3** [20], Cholesteryl-linked crown ether (transfection activity in HEK293 cells) **4** [21], Anilino-quinazoline-linked crown ether (EGFR inhibition) **5** [22], Fusidic acid (antiplasmodial activity) **6** [23].

Natural motifs and their synthetic derivatives are indeed significant for regulating biological systems [14,15]. Accordingly, their complexation in the crown scaffold has already gained considerable biomedical interest [16]. In this context, biologically active natural scaffold-linked crown ethers have been prepared and biologically investigated recently [17] (Figure 1).

**Scheme 1:**
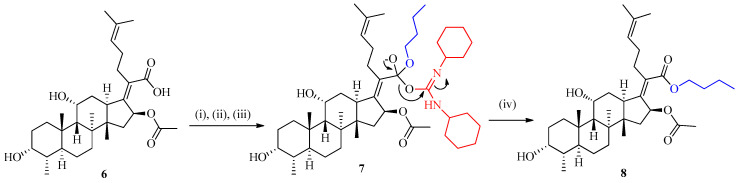
Preparation of Fusidic acid C-21 butyl ester. Reaction conditions: (i) Dimethyl amino pyridine, dichloromethane, 0 °C, 30 min; (ii) Dicyclohexylcarbodiimide, 0 °C, 1 h; (iii) 1-butanol, 0 °C, 3 h; (iv) 15 h at room temperature, then 15 h at 90 °C.

**Scheme 2:**
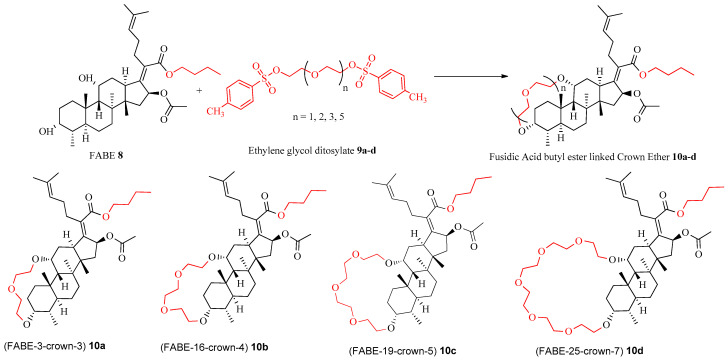
Preparation of Fusidic acid butyl ester-linked crown ethers. Reaction conditions: (a) K_2_CO_3_, DMF, 90 °C, 15 h.

Fusidic acid is a natural triterpene and a clinical medicine for various illnesses that include Clostridium difficile colitis, respiratory problems, cystic fibrosis, surgical prophylaxis, leprosy, as well as ophthalmic and neurological conditions [24], bacterial conjunctivitis [25], bone and joint infections [26,27,28], and soft-tissue infections [29,30]. However, the literature shows that Fusidic acid suffers from a narrow spectrum of biological properties and thus requires structural modification to expand its scope and potentially introduce new pharmacology [31,32]. For this purpose, various modification sites of Fusidic acid were focused, including C-3, C-11, C-16, C-17, C-20, C-21, C-24, and C-25, which often led to derivatives with comparatively higher biological activities. In this domain, various Fusidic acid C-21 ester derivatives were established to have meaningful antibacterial, antitumor, and anti-parasitic outcomes [33,34]. Motivated by the significance of crown ethers in the field of medicine and clinical effectiveness of Fusidic acid **6** (Figure 1 and Scheme 1) and its derivatives, we designed Fusidic acid C-21 butyl ester (FABE) **8** linked crown ethers of the type **10** (Scheme 2), to improve or add new pharmacology to Fusidic acid. This study focused on the novel modification of hydroxyl groups present at C-3 and C-11 positions of C-21 Fusidic acid butyl ester (FABE **8**, Scheme 2) by linking various flexible crown ether chains, and on discovering the biological properties of the resulting crown scaffolds.

In this paper, we report the preparation of novel crown ethers, as shown in Scheme 2, namely (FABE-13-crown-3) **10a**, (FABE-16-crown-4) **10b**, (FABE-19-crown-5) **10c**, and (FABE-25-crown-7) **10d**, having FABE **8** (Scheme 1) as a side arm, by attaching flexible crown ether chains at C-3 and C-11 hydroxyl groups of the FABE **8**. The modification in the ring size is made to create potency, selectivity, and tuning of biological properties. The structure of the prepared crown ethers was confirmed by ^1^HNMR, ^13^CNMR DEPT-135, FT-IR, and mass spectrometry. Moreover, we also report the preliminary evaluation of an antioxidant and α-glucosidase inhibition. Results obtained from the current studies indicated the promising antioxidant potential of all synthesized crown molecules except (FABE-13-crown-3) **10a** as compared to the standard butylated hydroxyanisole. Moreover, synthesized crown molecules exhibited moderate α-glucosidase inhibition activity against acarbose. In silico molecular docking investigations were performed using human antioxidant protein, peroxiredoxin 5, to validate the excellent experimental antioxidant potential of synthesized molecules, which demonstrated strong binding affinities of the prepared crown ether scaffolds, signifying satisfactory interactions at the molecular level. We also report the calculation of drug likeness properties that include physicochemical parameters, absorption, distribution, metabolism, excretion, and toxicity (ADMET) to evaluate the potential of the designed/prepared novel crown ethers, for consideration as drug candidates. ADMET analysis showed suitable pharmacokinetic and toxicity values, confirming the drug-likeness and therapeutic potential of these compounds. These results are comprehensively discussed in this paper. These experimental biological activities and in silico ADMET studies showed that the prepared novel FABE-linked crown ethers have promising molecular characteristics for future research to explore biological targets.

## 2. Results and Discussion

### 2.1. Chemistry

Synthesis of the designed crown ethers, **10a**–**d** was achieved in two steps as shown in Scheme 1 and Scheme 2. As a first step, Fusidic acid C-21 butyl ester (FABE) **8** was prepared using commercially available Fusidic acid **6** under modified Steglich conditions as shown in Scheme 1. As the second step, FABE **8** was used to prepare designed crown ethers by linking with ethylene glycol ditosylate **9a**–**d** in the presence of dimethylformamide as solvent and potassium carbonate as base at 90 °C, as shown in Scheme 2. We also prepared crown ethers **10a**–**d** with an alternative approach, with shortened reaction time and better yields.

#### 2.1.1. Preparation of Fusidic Acid C-21 Butyl Ester (FABE) **8**

Fusidic acid **6** was subjected to conversion into the corresponding Fusidic acid C-21 butyl ester (FABE) **8** by applying Steglich conditions, as shown in Scheme 1. Steglich esterification [35,36] is a well-reputed method for the preparation of esters under mild conditions [37]. The mildness of this reaction has been particularly valuable in synthesizing/modifying natural products [38,39]. To convert Fusidic acid **6** into butyl ester **8**, we employed reaction conditions that included coupling agent dicyclohexylcarbodiimide (DCC) with a catalytic amount of dimethylaminopyridine (DMAP) in dichloromethane as solvent at 0 °C (Scheme 1). As per the stepwise procedure, Fusidic acid **6** was stirred with dimethylaminopyridine (DMAP, 0.4 equiv.) in dichloromethane at 0 °C for 30 min (step i), allowing abstraction of the acidic proton. After that, N, N-dicyclohexylcarbodiimide (1.2 equiv.) was added and stirred at the same temperature (step ii). That allowed the carboxylate anion to add on carbodiimide to form O-acylurea (a known intermediate) [40]. After the complete formation of the intermediate during 1 h (based on TLC plate monitoring), 1-butanol (excess) was added and stirred for 3 h (step iii). That allowed effective addition of butanol to the O-acylurea and the formation of intermediate **7**. After that, the temperature was allowed to increase to room temperature and kept stirring for 15 h. This resulted in product **8** in lower yields (10% yield after column chromatography) along with the leftover intermediate **7**. Directed by this observation, the temperature was increased during step (iv) up to 90 °C for further 15 h, which provided 60% of the desired product (FABE) **8** after column chromatography.

This stable intermediate **7** was purified by silica gel column chromatography using a mixture of dichloromethane and methanol as eluent. The intermediate was characterized by ESI-MS and ^1^HNMR analysis. The ESI-MS analysis showed a molecular ion peak at *m*/*z* [M + H]^+^ = 797.7 calcd. 796.7 for C_48_H_80_N_2_O_7_. Further, in ^1^HNMR, the presence of absorption peaks in the range of (δppm = 1.11–0.8) corresponding to dicyclohexyl rings confirmed the structure of the isolated intermediate.

#### 2.1.2. Preparation of Fusidic Acid C-21 Butyl Ester-Linked Crown Ethers **10a**–**d**

Preparation of crown ethers **10a**–**d**, linked with FABE **8**, is shown in Scheme 2. The preparation of these crowns was made by the reaction involving FABE **8** and ethylene glycol ditosylate **9a**–**d**, under a sealed vessel, N_2_ atmosphere and in the presence of powdered K_2_CO_3_ and by employing oil bath heating at 90 °C in a 50 mL round bottom flask, following the literature protocol [41]. However, the reaction took 15 h to complete. This protocol resulted in crown ethers (38–62%) yields after column chromatography using silica gel as the stationary phase, and a mixture of dichloromethane and methanol (97:3) as eluent. The reaction provided crown ethers (FABE-13-crown-3) **10a**, (FABE-16-crown-4) **10b**, (FABE-19-crown-5) **10c**, and (FABE-25-crown-7) **10d** as shown in Scheme 2. The attempt to make crown ether (FABE-22-crown-6) using pentaethylene glycol ditosylate was unsuccessful and resulted in unidentified products. All prepared crown ethers were structurally characterized by NMR spectroscopic techniques along with mass spectrometry.

#### 2.1.3. An Alternate Approach for the Synthesis of Fusidic Acid Butyl Ester-Linked Crown Ether **10a**–**d**

To reduce the reaction time, we also discovered an alternate approach for the synthesis of crown ethers **10a**–**d** that relied on the in situ formation of butyl ester during the second step of the reaction sequence. In this alternate methodology, the 15 h of heating at 90 °C during step (iv, Scheme 1) were skipped, and only after 2 h of heating at 90 °C, the reaction mixture was worked up. After column chromatography using a mixture of dichloromethane and methanol (95:5) as eluent, solely intermediate 7 was isolated. Intermediate 7 was then reacted with ethylene glycol ditosylate **9a–d** under a sealed vessel, N_2_ atmosphere_,_ in the presence of powdered K_2_CO_3,_ by employing oil bath heating at 90 °C for 15 h in a 50 mL round bottom flask. This approach provided crown ethers **10a**–**d** in better yields (65–70%) after column chromatography using silica and a mixture of dichloromethane and methanol (97:3) as eluent.

### 2.2. Spectral Characterization

#### 2.2.1. ^1^HNMR Spectral Analysis

**Figure 2:**
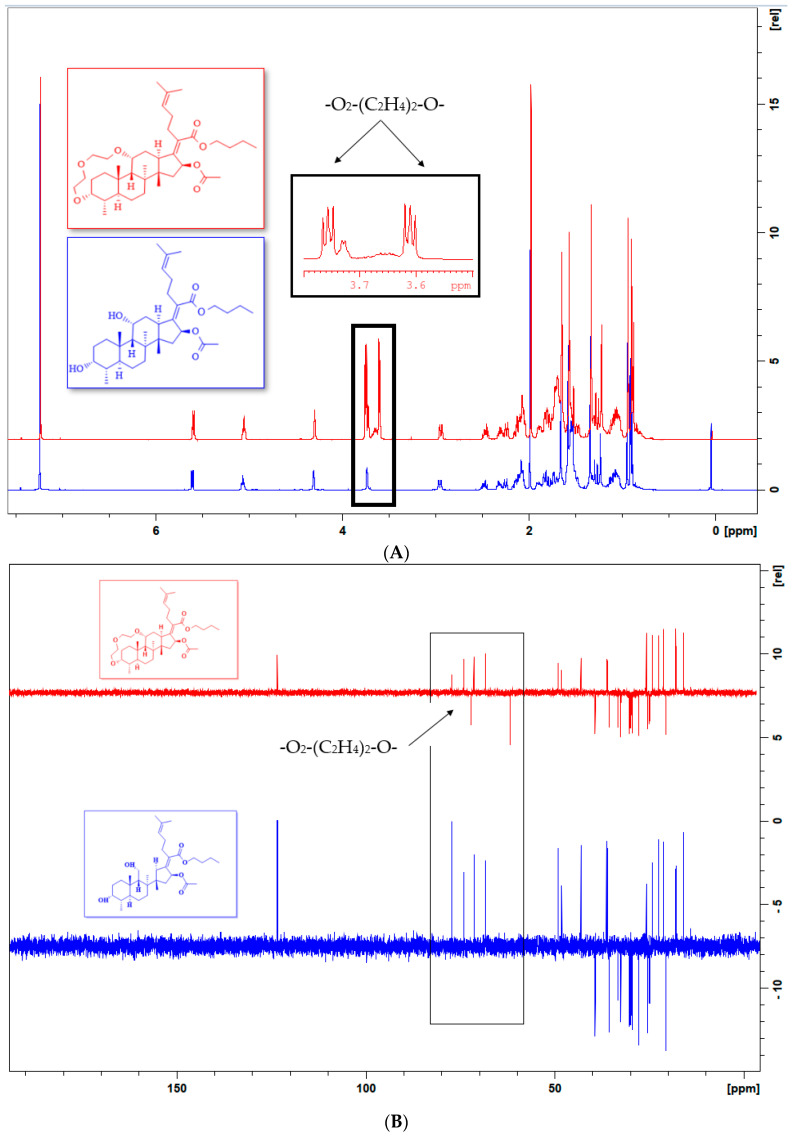
(**A**) Comparative ^1^HNMR of FABE (**8**, blue) and FABE 13-crown 3 (**10a**, red), (**B**) Comparative ^13^C-NMR of FABE (**8**, blue) and FABE 13-crown 3 (**10a**, red).

The most significant evidence for crown formation appeared in ^1^HNMR spectrum of compounds **10a**–**d**, that showed signals, apart from characteristic signals of FABE **8**. The comparative analysis of the ^1^HNMR spectrum showed absorption peaks corresponding to crown moiety -O_n_-(C_2_H_4_)_n_-O- linkages in the range of (δ = ppm) 4.26–3.54 as multiplet for 8H, 12H, 16H, and 24 H for (FABE-13-crown-3) **10a**, (FABE-16-crown-4) **10b**, (FABE-19-crown-5) **10c**, and (FABE-25-crown-7) **10d**, respectively, while recorded in CDCl_3_ at 500 MHZ (See Supplementary Materials). The comparative ^1^HNMR spectra of representative crown (FABE-13-crown-3) **10a** and FABE **8** are shown in Figure 2A. The comparative spectral analysis showed the absorption peaks corresponding to 8H for -O_2_-(C_2_H_4_) _2_-O- in the region of 4.26–3.54 ppm for the spectrum of **10a** (shown in red), which were absent in the spectrum of **8** (shown in blue). 

#### 2.2.2. ^13^C NMR Spectral Analysis

^13^C NMR DEPT-135 spectrum recorded at 400 MHz in CDCl_3_ of all the crown ether molecules **10a**–**d** indicated all the characteristic signals of FABE **8** along with the negative absorption peaks corresponding to -O_n_-(C_2_H_4_)_n_-O- linkages of the crown chain between the range (δ = ppm) 75.61–61.90. The comparative ^13^C NMR DEPT-135 spectra of representative crown **10a** with FABE **8** are shown in Figure 2B. The comparative spectral analysis showed the negative absorption peaks corresponding to 4CH_2_ groups for -O_2_-(C_2_H_4_) _2_-O- in the region of 75.61–61.90 ppm for the spectrum of 10a (shown in red), which were absent in the spectrum of 8 (shown in blue).

#### 2.2.3. FT-IR Spectral Analysis

FT-IR spectroscopy was performed for precursor FABE **8** and the crown molecules **10a**–**d** in methanol solution in the ATR mode. The FT-IR spectrum recognized various functional groups of the crown ether molecules. Careful comparison of the FT-IR spectrum of crown **10a**–**d** with FABE **8** showed the presence of many weak signals in the range of ν(cm^−1^) 1280–1000, corresponding to C-O-C fragments, which were absent in the FT-IR spectrum of Fusidic acid butyl ester (FABE) **8**. Further, the absorption at ν(cm^−1^) 2925–2857 corresponding to CH_2_ stretching intensified compared to FABE **8**. It is important to note that the broadness of the band ν(cm^−1^) at 3400 was increased in the FT-IR spectrum of the crown molecule (dissolved in methanol), which showed the intermolecular hydrogen bonding of the oxygen moiety of the crown ether with the methanol [42] (See Supplementary Materials).

#### 2.2.4. ESIMS Analysis

**Figure 3:**
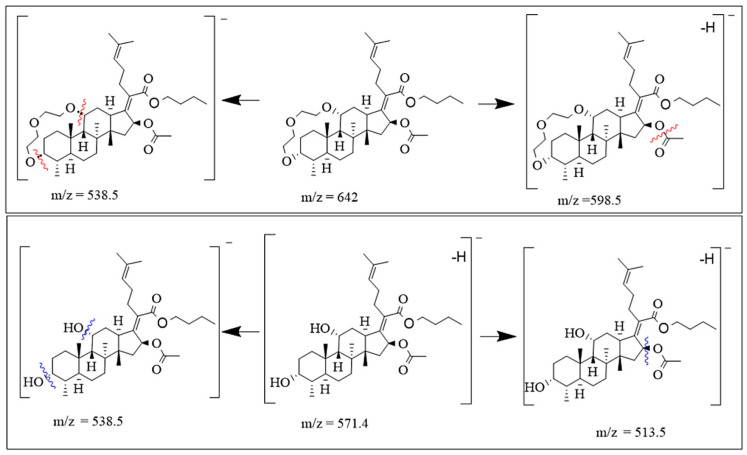
ESIMS fragmentation of **10a** and precursor **8**.

Electrospray ionization mass spectrometry (ESIMS) is a soft ionization technique that enables most of the molecules to be analyzed without significant structural changes. The ESIMS method was used to confirm the molecular masses of prepared crown molecules (dissolved in methanol), which resulted in the formation of common fragments, for example, M-H, M-COCH_3_, M-OCOCH_3_, and M-{(O-C_2_H_4_)_n_-O}, where M is the molecular mass of the crown **10a**–**d** (Scheme 2). The formation of fragment ion with the loss of -2OH groups corresponding to [C_35_H_54_O_4_] at *m*/*z* = 538.5 is the most stable ion that appeared as base ion peak in the spectrum of precursor, i.e., FABE **8** and also appeared in the spectrum of crown molecules generally, at *m*/*z* = 538.5 as a base peak with the loss of crown chain -O_n_-(C_2_H_4_)_n_-O-. Further fragmentation with the loss of acetyl, i.e., M-COCH_3_, or loss of acetyloxy, i.e., M-OCOCH_3_, is another most common fragmentation pattern that was observed in the ESIMS spectrum of Fusidic acid butyl ester and of all crown molecules. The comparative ESIMS analysis of crown (FABE-13-crown-3) **10a**, with FABE 8 and the primary fragment ions generated under ESI conditions are shown in Figure 3.

### 2.3. Biological Studies

#### 2.3.1. Antioxidant Activity Investigations

Oxidative stress is an imbalance between in vivo generated reactive oxygen/ nitrogen species (ROS/NOS) and the cellular antioxidant defenses [43]. Oxidative stress is connected with more than one hundred illnesses that include cardiovascular, cancer, neurogenerative diseases, hypertension, atherosclerosis, and diabetes mellitus [44]. Antioxidants decrease the cellular level of ROS and thus play a significant role in preventing related diseases. Antioxidants react with ROS through three possible mechanisms: (i) hydrogen transfer, (ii) electron transfer, and (iii) metal chelation. DPPH radical scavenging assay relies on the electron/hydrogen atom transfer (mixed) mechanism [45]. The prepared crown ethers with variable cavity sizes **10a**–**d**, parent precursor (FABE) **8**, and the novel intermediate **7** were examined for antioxidant behavior using the DPPH radical scavenging protocol [46], following the literature, and the results are shown in Table 1. The antioxidant potential of these compounds was compared with that of butylated hydroxyanisole (BHA) as a standard (IC_50_ = 44.2 ± 0.4 μM). Remarkably, the crown molecules exhibited outstanding antioxidant activity (IC_50_ = 22.5 ± 0.2 to 45.4 ± 0.9 μM) except (FABE-13-crown-3) **10a**, having the smallest cavity size, which was found to be the least active (IC_50_ = 65.5 ± 0.3 μM) among all. (FABE-19-crown-5) **10c** was found the most active compound with (IC_5_ 22.5 ± 0.2 μM) followed by (FABE-16-crown-4) **10b** with (IC_50_ 32.1 ± 0.3 μM) in this study. Antioxidant activity of crown (FABE-25-crown-7) **10d** was comparable (IC_50_ = 45.4 ± 0.9 μM) with the standard BHA. These results showed the suitability of the cavity size of (FABE-19-crown-5) **10c** and (FABE-16-crown-4) **10b** for this activity. Further, it was observed that the crown molecules exhibited greater antioxidant potential as compared to parent Fusidic acid C-21 butyl ester **8** (IC_50_ = 54.6 ± 0.2 μM, Table 1), which suggested the importance of the crown moiety {(-O-CH_2_CH_2_)_n_-O} for this activity, which might be recognizing their radical chelation abilities.

#### 2.3.2. α-Glucosidase Inhibition Investigations

Diabetes mellitus stands third among the death causing diseases around the world and is characterized by inadequate glucose levels in the blood. Among the core abnormalities associated with diabetes mellitus, the most important one is oxidative stress. Biochemical investigations in diabetic patients have shown an increased production of reactive oxygen species in the tissues and cells [47]. Antioxidants are generally believed to be better antidiabetic agents [48,49]. In this context, α-glucosidase inhibitors are clinically approved drugs for treating diabetes type-2 patients [50]. In this background, according to the standard protocol [51], we examined the prepared crown ether molecules **10a**–**d** along with parent precursor FABE **8** and the novel intermediate **7** for their in vitro α-glucosidase inhibition potential. The results are shown in Table 1. All the crown molecules showed a moderate level of α-glucosidase inhibition within the range of (IC_50_ = 23.5 ± 0.2 to 65.5 ± 0.3 μM) when compared to the standard acarbose (IC_50_ = 5.2 ± 0.8 μM, Table 1). Interestingly, (FABE-19-crown-5) **10c**, the most potent antioxidant, was also found to be the most active compound with (IC_50_ = 23.5 ± 0.2 μM) for this assay. However, the crown ethers showed generally reduced inhibitory potential as compared to the parent precursor FABE **8** (IC_50_ = 43.2 ± 0.8 μM).

### 2.4. Molecular Docking Investigations

The in silico molecular docking studies were performed to further support the results of in vitro antioxidant activity [52]. For this study, the three-dimensional crystalline structure of human antioxidant protein, peroxiredoxin 5 (PRDX5), was retrieved from the standard source Protein Data Bank (RCSB: PDB) with PDB ID: 1HD2 at 1.5 Å resolution, by using the standard protocol [53]. Peroxiredoxin 5 (PRDX5) is a human thioredoxin peroxidase widely found in cells and tissues. It is concerned with antioxidant defensive mechanisms.

All ligands (crown ethers **10a**–**d** and FABE **8**) showed significant binding in the binding active site of human peroxiredoxin 5. The experimental outcomes indicated that **10b** and **10c** were the most potent crown ethers, while **10a** analog was the least active. To see the general binding trends, we docked all crown ethers and the precursor ester **8** into the target protein and compared them with the standard antioxidant drug BHA.

**Figure 4:**
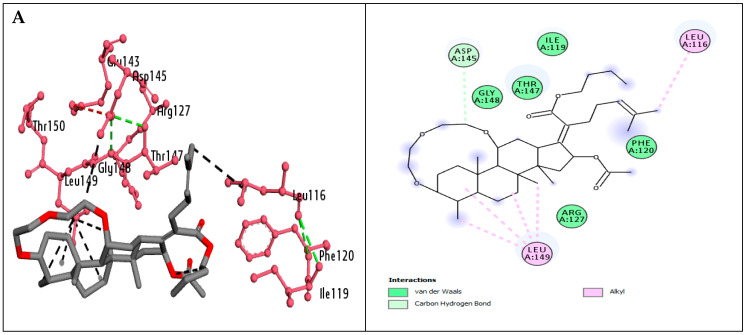

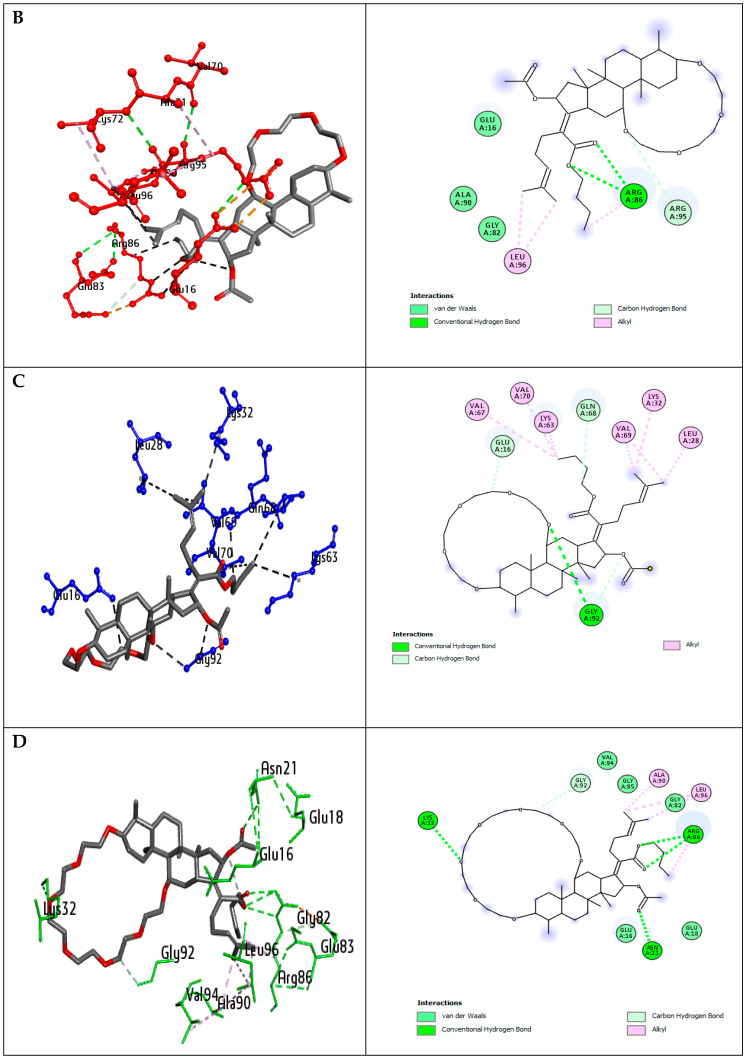

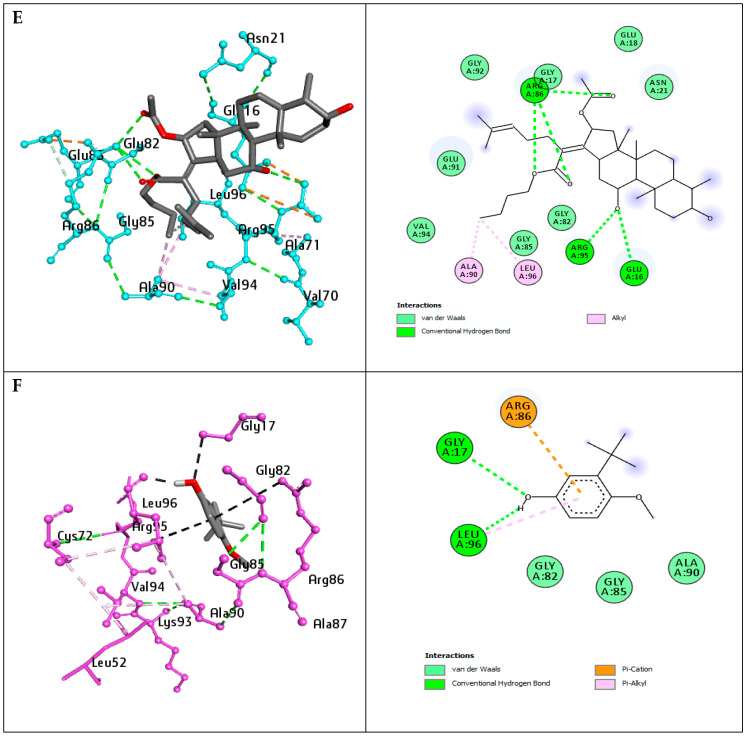
(**A**–**F**): Binding interactions of compound **10a** (**A**), **10b** (**B**), **10c** (**C**), **10d** (**D**), **8** (**E**), and standard compound BHA (**F**) against human antioxidant protein peroxiredoxin 5.

Figure 4 shows the binding interactions obtained by molecular docking of crown ethers into the human peroxiredoxin 5, while Table 1 shows the binding affinities. The amino acid residues of the binding active site of human peroxiredoxin 5 showed conventional hydrogen bonding, alkyl bond, and carbon–hydrogen interactions with the crown ether compounds.

All the docked crown ether compounds showed the same pattern of alkyl bond interactions involving C-26 and C-27 with Leu A:96 or Leu A:28. Generally, these weak interactions of alkyl bonds typically support the attachment of the ligands to the binding site of the receptor and contribute to the stabilization of receptor–ligand interaction. It is important to note that the experimentally most active compound (FABE-19-crown-5) **10c** showed the highest number of alkyl bond interactions (06) involving C-26, C-27 with Lys A:32, Val A:69, and Leu A:28; and involving CH_3_ group of –COOC_4_H_9_ interacting with Lys A:63, Val A:69, and Val A:70. Interestingly, (FABE-19-crown-5) **10c**, showed the least number of conventional hydrogen bond interaction (01), involving oxygen of crown chain with Gly A:92. Rest of the interactions are three (03) carbon hydrogen interactions involving CH_2_ of –COOC_4_H_9_ with Gln A:68, oxygen of CH_3_COO- with Gly A:92, and carbon of crown chain with Glu A:16. These interactions showed the importance of butyl ester fragment and the crown ether moiety for the binding with protein. Similarly, the second most active compound (FABE-16-crown-4) **10b** showed only two conventional hydrogen bond interactions and 03 alkyl bond interactions (see Supplementary Materials). All other molecules with more conventional hydrogen bond interactions have been found experimentally to be less active. This pattern suggested that increased hydrogen bonds could significantly enhance the binding affinity between the molecule and the protein. Such strong interactions may lead to a more stable complex, inhibiting the molecule’s ability to respond effectively. Understanding this relationship is crucial for developing strategies to overcome resistance mechanisms in drug design.

### 2.5. ADME-T Result Interpretation

In silico ADMET parameters (absorption, distribution, metabolism, excretion, and toxicity for Fusidic acid **6**, reaction intermediate **7**, precursor (FABE) **8**, and the crown molecules **10a**–**d** were determined by using online tool (SwissADME and pkCSM) [54,55]. We performed in silico ADMET studies to predict molecular weights (MW), octanol/water partition coefficient (LogPo/w), number of hydrogen bond donor atoms (HBD), number of hydrogen bond acceptor (HBA), topological polar surface area (TPSA), and the number of rotatable bonds (RotB), and results are shown in Table 2. Prediction of drug likeness is one of the ways to explore the pharmacology of a potential drug candidate [56,57]. There are several descriptors for molecules to predict drug likeness, for example, rule of five (RO5), also known as Lipinski’s rule of five, which explains the chemical as “likely to be oral” [58]. However, beyond RO5 (bRO5) describes a significant extension of the traditional RO5 chemical space to a markedly bigger “possible to be oral”, which suggests that the limit for oral bioavailability extends to approximately MW ≤ 1000 Da, −2 ≤ LogPo/w ≤ 10, HBD ≤ 6, HBA ≤ 15, TPSA ≤ 250 Å^2^, and RotB ≤ 20. Natural products and their derivatives are the key sources of bRO5 chemical space [59,60].

All the predicted values found within range, for example, MW (642.91–805.09), LogPo/w (5.96–7.16), HBD (0), HBA (7–11), TPSA (80.29–117.21 Å^2^) and RotB (10). Thus, prepared crown obeyed bRO5 and showed suitability for oral bioavailability.

TPSA is a parameter that predicts the transport characteristics of the compound [61]. For optimal oral bioavailability, the value of TPSA must lie within the range of 20 to 130 Å^2^. All prepared crown ethers found with computational TPSA (80.29–117.21 Å^2^), indicating good oral potential. The number of RotB indicates the flexibility of the molecules and suggests the ease of binding with biological receptors. All studied crown ethers exhibited 10 number of rotatable bonds that predicted the good binding abilities with the protein receptors, indicating good oral bioavailability potential.

Our designed crown ethers also followed the Veber’s rule, which states “to present good bioavailability, a chemical substance must have number of rotatable bonds no greater than 10 and a topological polar surface area (TPSA) no greater than 140 Å^2^”.

Absorption of a drug molecule can be determined by physiochemical parameters such as aqueous solubility (can be measured by log Sw), lipophilicity (log Po/w), Human intestinal absorption (HIA %), and permeability parameters (such as the coefficient of permeability Kp, Caco-2, and Blood–Brain Barrier, i.e., BBB) values [62]. We also performed in silico ADMET analysis to predict aqueous solubility, Human intestinal absorption, the coefficient of permeability, Caco-2, Blood–Brain Barrier, P-gp substrate, cytochrome P450, and the results were summarized in Table 3.

The synthesized crown compounds are expected to be moderately soluble in water, with calculated values of the solubility, log Sw in the range of (−3.573 to −3.79 mol/L). On the other hand, calculated, higher values of partition coefficient, log P o/w (n-octanol and water), shown in Table 3 for synthesized analogs (5.96–7.16), indicated that the lipophilicity of all crown adducts is significantly improved as compared to Fusidic acid and its butyl ester. Further log Po/w provides an indirect impression about the penetration and permeability of drug candidates. The coefficient of skin permeability (logKp) of all synthesized compounds are in the range of −2.7 (cm/s), that predicted good skin penetration (recommended value of log Kp should be −2.5 cm/s for better efficacy and transdermal delivery of the drug). Caco-2 permeability, which measures the absorption in intestinal mucosa, was found in accordance with their potential to act as expected drug candidates. Human intestinal absorption (HIA) is an important parameter, as most of the drugs administrated orally are more likely to be absorbed in the small intestine [63]. Interestingly, all crown adducts and the ester precursor showed excellent absorption ranges (calc. 99.2 to 100%). Notably, the calculated values of HIA are improved in crown adducts **10a**–**d** and in **8** as compared to the Fusidic acid itself.

Parameters such as the blood–brain barrier (BBB/log BB) and P-gp substrate are usually used to express drug distribution. The predicted values of the synthesized analogs were found in the range of −1.49 to +0.07 (standard range for log BB is −3.0 to +1.2), which showed the good distribution potential of the compounds.

P-glycoprotein (P-gp) is an energy-driven efflux transporter protein widely distributed in the body such as intestine, skin cells, biliary duct, liver, and kidney. P-gp is responsible for preventing the absorption of foreign bodies in the intestine. P-gp inhibitors are believed to increase drug absorption, whereas P-gp substrates decrease drug absorption by being expelled from the cells when co-administrated [64]. Therefore, predicting whether a compound is a P-gp substrate or an inhibitor becomes critical in early drug development stages. All crowns were found to be non-substrate P-gp while expressing their P-gp inhibitor potential. Therefore, these crown compounds showed their potential to be used as efflux inhibitors (P-gp inhibitors) [65,66], which is considered an effective strategy for better efficacy of antimicrobial drugs. Cytochrome P450 (CYP450) is a membrane-bound protein that controls the drug metabolism and, in turn, the length of a drug’s stay in the body [67]. The inhibitors of CYP450 are utilized to minimize drug metabolism and toxicity. All the examined compounds were predicted to be CY3A4 inhibitors.

Inhibition of CY3A4 can enhance the effectiveness of therapeutic agent of the swiftly metabolized drugs by increasing their plasma levels. Genetic toxicity (AMES toxicity) and impairment of liver function (hepatotoxicity) parameters also predicted the nontoxic nature of these crowns (Table 3).

## 3. Materials and Methods

### 3.1. General

All organic reagents and chemicals (1-butanol, dichloromethane, methanol, acetonitrile, and dimethyl formamide, Fusidic Acid, dicyclohexylcarbodiimide, dimethyl aminopyridine, and diethylene glycol ditosylate) and all other supplies were purchased from Sigma Aldrich and Merck. Chemicals were used without any further purification. Cleaned and dried glass wares were used for the experimentation. Silica gel (0.063–0.200 mm) was used for column chromatography. Silica gel (0.063–0.200 mm, 70–230 mesh; ASTM, Merck, Darmstadt, Germany) was used for column chromatography. To monitor reaction completion and purification, commercially available silica gel plates (Merck, Merck, Darmstadt, Germany, F254) were used for thin layer chromatography, and spots were visualized by UV Light at λ = 254 nm. For the spectral data of synthesized compounds ^1^H NMR, ^13^C NMR DEPT-135, ESI-MS, and FT-IR were used. ^1^H NMR, ^13^C NMR DEPT-135 were recorded on an Avance NEO spectrometer at 500 MHz, in CDCl_3_ at room temperature with tetramethyl silane (TMS) used as internal standard. Coupling constant (J Values) and chemical shifts were reported in Hz and ppm, respectively. ESI-MS spectra in negative mode were recorded on Bruker Compass Data Analysis 4.2. FTIR spectra were recorded on a Shimadzu IR-460 in the 400–4000 cm^−1^ range. Online web tools (Swiss ADME and pkCSM) were used to analyze ADMET studies.

### 3.2. Synthetic Approach

#### 3.2.1. Synthetic Procedure for the Synthesis of Fusidic Acid Butyl Ester 8

The stirred solution of 0.20 mmol of Fusidic acid in 6.0 mL anhydrous dichloromethane (CH_2_Cl_2_) and dimethyl aminopyridine (10 mg, 4 equiv., 0.80 mmol) was kept in an ice bath at 0 °C for 30 min. Afterwards, dicyclohexyl carbodiimide (10 mg, 1.2 equiv. 0.24 mmol) was added and stirred at 0 °C for 1 h. Then, 1 butanol (excess) was added, and the mixture was stirred for 3 h. Afterwards, it was stirred at room temperature for 15 h. After that, the reaction mixture was heated at 50–55 °C for 3 h and then at 90 °C for 15 h. The reaction mixture was quenched with 5.0 mL of water and extracted with dichloromethane twice, and the combined organic layers (CH_2_Cl_2_) were dried under vacuum. A total of 1.0 mL of acetonitrile was added, which dissolved the crude product, leaving insolubilized dicyclohexyl urea removed by filtration (twice). The organic solvent was evaporated by vacuum, and crude Fusidic butyl ester (**8**) was purified in 60% yield by short silica gel column chromatography, using 99:1 chloroform:methanol as mobile phase.

##### Butyl(2Z)-2-[(4a,8a,14b,16b)-16-(acetyloxy)-3,11-dihydroxy-4,8,10,14-tetramethylgonan-17-ylidene]-6-methylhept-5-enoate (**8**)

Yield (52%); FT-IR, KBr, cm^−1^: v(O-H) 3330 cm^−1^, ν (C-H stretching) 2928–2852 cm^−1^, ν (C=O, Ester) 1734 cm^−1^, stretching at 1626 cm^−1^ corresponds to C=C bond. ν (C-H, scissoring) 1449 cm^−1^, ν (methyl rocking) at 1373 cm^−1^, ν (=C-H bend) at 1091 cm^−1^. HNMR (500 MHz, CDCl_3_), δ/ppm: 0.89 (d, 3H, *J* = 7 Hz, C28-CH_3_),0.91 (s, 3H, C18-CH_3_), 0.94 (s, 3H, C19-CH_3_), 1.01–1.16 (m, 5H, C7-H, C6-H, butyl-3H), 1.23 (s, 2H, butyl-2H), 1.26 (d, 1H, *J* = 14 Hz, C15-H), 1.313 m, 1H, butyl-H), 1.34 (s, 3H, C29-CH3), 1.47–1.50 (m, 1H, C1-H), 1.51 (m, 2H, butyl-1H, C9-H), 1.53 (s, 3H, C26-CH_3_), 1.61 (m, 1H, C4-H), 1.65 (s, 3H, C27-CH_3_ ), 1.65–1.83 (m, 6H, C6-H, C7-H, C2-2H, butyl-2H), 1.83–1.86 (m, 1H, C12-H), 1.94 (s, 3H, C31-CH3), 2.00–2.13 (m, 5H, C1-H, C5-H, C23-2H, C22-1H), 2.18–2.22 (m,1H, C15-H), 2.24–2.30 (m, 1H, C12-H), 2.40–2.46 (m, 1H, C22-H) 2.90 (d, 1H, *J* = 12 Hz, C13-H), 3.7 (m, 1H, C3-H), 4.303 (m, 1H, Hz, C11-H), 5.04 (m, 1H, *J* = 7.5 Hz, C24-H), 5.58 (d, 1H, *J* = 8.20 Hz, C16-H). ^13^CNMR DEPT-135, 500 MHz, CDCl_3_), δ/ppm = 15.92 (CH_3_), 17.84 (CH_3_), 17.96 (CH_3_), 20.66 (CH_2_), 21.22 (CH_3_), 22.52 (CH_3_), 24.27 (CH_3_), 25.74 (CH_3_), 27.84 (2CH_2_), 29.48 (CH_2_), 29.98 (CH_2_), 30.35 (CH_2_), 32.55, (CH_2_), 32.64 (CH_2_), 33.34 (CH_2_), 35.61 (CH_2_), 36.03 (CH), 36.34 (CH), 39.30 (CH_2_), 43.11 (CH), 48.18 (CH), 49.15 (CH), 68.38 (CH), 71.35 (CH), 74. 08 (CH), 123.40 (CH), ESIMS (*m*/*z*): 571.4 = M-H, fragment ion 538.5 corresponds to M-2OH, 513.5 [M−OCOCH_3_].

#### 3.2.2. Synthetic Procedure for the Synthesis of Fusidic Acid Butyl Ester-Linked Crown Ethers

The solution of 0.20 mmol of Fusidic acid butyl ester in 6.0 mL anhydrous dimethyl formamide (DMF) and K_2_CO_3_ (10 mg, 4 equiv.) was stirred for 1 h at room temperature under a nitrogen atmosphere. Afterwards, the reaction mixture was heated at 90 °C for 19 h while continuously stirring under a nitrogen atmosphere. The reaction mixture was dried under vacuum and then subjected to column chromatography using (97:3) chloroform:methanol as mobile phase. Purified crown ether was obtained with a 60% yield by short silica gel chromatography.

##### Butyl(2Z)-2-[(3aS,16S,17aS,17bS,18S)-16-(acetyloxy)-3a,17a,17b,18-tetramethyloctahydro-3,6 methanocyclopenta[3,4]naptho[1,8hi][1,4,7]trioxyacylotridecin-15(1H)-ylidene]-6-methylhept-5-enoate (FABE-13-crown 3) **10a**

Yield (35%); FT-IR (ATR) cm^−1^: ν (Intramolecular Hydrogen Bonding) 3320.01 cm^−1^, ν (C-H stretch) 2927.51–2855.66 cm^−1^, ν(C=O, ester) 1743.49 cm^−1^, ν(C=C) 1627.59 cm^−1^, ν(C-O) 1374.61 cm^−1^, ν (C-O-C stretch) 1258.33 cm^−1^, ν(=C–H bending) at 1029 cm^−1^. ^1^HNMR (500 MHz, CDCl_3_), δ/ppm: 0.84 (d, 3H, *J* = 7 Hz, C28-CH_3_), 0.86 (s, 3H, C18-CH_3_), 0.90 (s, 3H, C19-CH3), 0.99–1.07 (m, 5H, C7-H, C6-H, butyl-3H), 1.18 (s, 2H, butyl-2H) 1.22 (d, 1H, *J* = 14 Hz, C15-H), 1.26–1.27 (m, 1H, butyl-H), 1.30 (s, 3H, C29-CH_3_), 1.43–1.45 (m, 1H, C1-H), 1.46–1.49 (m, 2H, butyl-1H, C9-H), 1.53 (s, 3H, C26-CH_3_), 1.56 (m, 1H, C4-H), 1.61 (s, 3H, C27-CH_3_), 1.69–1.80 (m, 6H, C6-H, C7-H, C2-2H, butyl-2H) 1.83–1.85 (m, 1H, C12-H), 1.94 (s, 3H, C31-CH_3_), 2.00–2.10 (m, 5H, C1-H, C5-H, C23-2H, C22-1H), 2.16 –2.23 (m,1H, C15-H), 2.23–2.30 (m,1H, C12-H), 2.35–2.37 (m,1H, C22-H), 2.94 (d, 1H, *J* = 12 Hz, C13-H), 3.56–3.65 (m, 5H, OC_2_H_4_-5H), 3.68 (m, 1H, C3-H), 3.69–3.75 (m, 3H, OC_2_H_4_-3H), 4.26 (m, 1H, Hz, C11-H), 5.00 (m, 1H, C24-H), 5.56 (d, 1H, *J* = 8.20 Hz, C16-H).^13^CNMR DEPT-135, 500 MHz, CDCl_3_), δ/ppm = 15.93 (CH_3_), 17.85 (CH_3_), 17.96 (CH_3_), 20.69 (CH_2_), 21.23 (CH_3_), 22.56 (CH_3_), 22.56 (CH_3_), 24.25 (CH_3_), 25.75 (CH_3_), 27.86 (2CH_2_), 29.50 (CH_2_), 29.99 (CH_2_), 30.36 (CH_2_), 32.62 (2CH_2_), 33.34 (CH_2_), 35.63 (CH_2_), 36.07 (CH), 36.33 (CH), 39.31 (CH_2_), 43.13 (CH), 48.20 (CH), 49.18 (CH), 61.78 (2CH_2_), 68.39 (CH), 71.37 (CH), 72.09 (2CH_2_), 74.10 (CH), 123.40 (CH), ESIMS (*m*/*z*): 598.5 = M-H-COCH_3_ and 538.5 = M-(CH_2_CH_2_)_2_O_3_

##### Butyl(2Z)-2-[(3aS,19S,20aS,20bS,21S)-19-(acetyloxy)-3a,20a,20b,21-tetramethylicosahydro-3,6-methanocyclopenta[3,4]naphtho[1,8-kl][1,4,7,10]tetraoxacyclohexadecin-18(1H)-ylidene]-6-methylhept-5-enoate (FABE-16-crown-4) **10b**

Yield (32%); FT-IR (ATR) cm^−1^: ν (Intramolecular Hydrogen Bonding) 3320.01 cm^−1^, ν (C-H stretch) 2925.84 -2854.39 cm^−1^, ν (C=O, ester) 1743.87 cm^−1^, ν(C=C) 1624.72 cm^−1^, ν(rocking C-H) 1452.49 cm^−1^, ν(C-O) 1374.90 cm^−1^, ν (C-O-C stretch) 1259.03 cm^−1^, ν(=C–H bending) at 1030 cm^−1^. ^1^HNMR (500 MHz, CDCl_3_), δ/ppm: 0.89 (d, 3H, *J* = 7 Hz, C28-CH_3_), 0.90 (s, 3H, C18-CH_3_), 0.91 (s, 3H, C19-CH_3_), 1.04–1.12 (m, 5H, C7-H, C6-H, butyl-3H), 1.23 (s, 2H, butyl-2H) 1.27 (d, 1H, *J* = 14 Hz, C15-H), 1.30–1.32 (m, 1H, butyl-H), 1.34 (s, 3H, C29-CH_3_), 1.47–1.50 (m, 1H, C1-H), 1.52 (m, 1H, butyl-1H), 1.55(m, 1H, C9-H), 1.57 (s, 3H, C26-CH_3_), 1.59 (m, 1H, C4-H), 1.65 (s, 3H, C27-CH_3_), 1.74–1.83 (m, 6H, C6-H, C7-H, C2-2H, butyl-2H) 1.87–1.91 (m, 1H, C12-H), 1.99 (s, 3H, C31-CH_3_), 2.08–2.16 (m, 5H, C1-H, C5-H, C23-2H, C22-1H), 2.24 –2.31 (m, 1H, C15-H), 2.28–2.33 (m, 1H, C12-H), 2.44–2.50 (m, 1H, C22-H), 2.95 (d, 1H, *J* = 12 Hz, C13-H), 3.41–3.99 (m, 13H, OC_2_H_4_-12H, C3-H), 4.30 (m, 1H, Hz, C11-H), 5.06 (m, 1H, C24-H), 5.60 (d, 1H, *J* = 8.20 Hz, C16-H). ^13^CNMR DEPT-135, 500 MHz, CDCl_3_), δ/ppm = 15.93 (CH_3_), 17.845 (CH_3_), 17.94 (CH_3_), 20.70 (CH_2_), 21.22 (CH_3_), 22.59 (CH_3_), 24.21 (CH_3_), 25.74 (CH_3_), 27.85 (2CH_2_), 29.48 (CH_2_), 29.97 (CH_2_), 30.33 (CH_2_), 32.56 (2CH_2_), 33.33 (CH_2_), 35.62 (CH_2_), 36.08 (CH), 36.28 (CH), 39.30 (CH_2_), 43.12 (CH), 48.19 (CH), 49.18 (CH), 61.75 (2CH_2_), 68.37 (CH), 70.40 (2CH_2_), 71.36 (CH), 72.54 (2CH_2_), 74.08 (CH), 123.39 (CH), ESIMS (*m*/*z*): 684.4 = M-2H and 538.5 = M-(CH_2_CH_2_)_3_O_4_

##### Butyl(2Z)-2-[(3aS,22S,23aS,23bS,24S)-22-(acetyloxy)-3a,23a,23b,24-tetramethyldocosahydro-3,6-methanocyclopenta[3,4]naphtho[1,8-no][1,4,7,10,13]pentaoxacyclononadecin-21(1H)-ylidene]-6- methylhept-5-enoate (FABE 19-crown-5) **10c**

Yield (37%); FTIR (ATR) cm^−1^: ν (Intramolecular Hydrogen Bonding) 3415.91 cm^−1^, ν (C-H stretch) 2930.36–2872.84 cm^−1^, ν (C=O, ester) 1717.50 cm^−1^, ν(C=C) 1555.83 cm^−1^, ν (rocking C-H) 1455.09 cm^−1^, ν(C-O) 1374.81 cm^−1^, ν (C-O-C stretch) 1252.74 cm^−1,^ ν (=C–H bending) at 1027.78 cm^−1^; 0.84 (d, 3H, *J* = 7 Hz, C28-CH_3_), 0.87 (s, 3H, C18-CH_3_), 0.90 (s, 3H, C19-CH_3_), 1.00–1.09 (m, 5H, C7-H, C6-H, butyl-3H), 1.18 (s, 2H, butyl-2H) 1.21 (d, 1H, *J* = 14 Hz, C15-H), 1.25 (m, 1H, butyl-H), 1.31 (s, 3H, C29-CH_3_), 1.43–1.46 (m, 1H, C1-H), 1.48 (m, 2H, butyl-1H, C9-H), 1.53 (s, 3H, C26-CH_3_), 1.55 (m, 1H, C4-H), 1.60 (s, 3H, C27-CH_3_), 169–1.78 (m, 6H, C6-H, C7-H, C2-2H, butyl-2H) 1.84–1.87 (m, 1H, C12-H), 1.90 (s, 3H, C31-CH_3_), 2.02–2.10 (m, 5H, C1-H, C5-H, C23-2H, C22-1H), 2.23–2.25 (m, 1H, C15-H), 2.34–2.41 (m, 1H, C12-H, C22-H), 2.95 (d, 1H, *J* = 12 Hz, C13-H), 3.21–3.70 (m, 13H, OC_2_H_4_-12H, C3-H), 3.72–4.26 (m, 4H, OC_2_H_4_-4H), 4.27 (m, 1H, Hz, C11-H), 5.02(m, 1H, C24-H), 5.75 (d, 1H, *J* = 8.20 Hz, C16-H). ^13^CNMR (Dept-135, 400 MHz, CDCl_3_), δ/ppm = 15.92 (CH_3_), 17.78 (CH_3_), 17.87 (CH_3_), 20.72 (CH_2_), 21.00 (CH_3_), 22.65 (CH_3_), 24.24 (CH_3_), 25.73 (CH_3_), 28.37 (CH_2_), 29.02 (CH_2_), 29.98 (CH_2_), 30.32 (CH_2_), 30.51(CH_2_), 32.54 (2CH_2_), 35.57(CH_2_), 36.15 (CH), 36.27 (CH), 39.03 (CH_2_), 43.81 (CH), 49.20 (2CH), 61.77 (CH_2_), 64.42 (CH_2_), 68.34 (CH), 70.34 (CH_2_), 70.53 (CH_2_), 70.67 (CH_2_), 71.36 (CH), 74.46 (CH), 123.16 (CH), ESI-MS (*m*/*z*): 684.3 = M-3H-COCH_3_ and 538.5 = M-(CH_2_CH_2_)_3_O_4_.

##### Butyl(2Z)-2-[(3aS,27S,28aS,28bS,29S)-27-(acetyloxy)-3a,28a,28b,29-tetramethyltetracosahydro-3,6-methanocyclopenta[3,4] naphtho[1,8-st][1,3,6,9,12,15,18]heptaoxacyclotetracosin-26(1H)-ylidene]-6- methylhept-5-enoate (FABE-25-crown-7) **10d**

Yield (56%); FT-IR (ATR) cm^−1^: ν (Intramolecular Hydrogen Bonding) 3320.61 cm^−1^, ν (C-H stretch) 2928.61–2856.31 cm^−1^, ν (C=O, ester) 1734.82 cm^−1^, ν (C=C) 1628.30 cm^−1^, ν(rocking C-H) 1451.32 cm^−1^, ν (C-O) 1320.17 cm^−1^, ν (C-O-C stretch) 1256.39–1237.60, ν (=C–H bending) at 1029.30 cm^−1^. ^1^HNMR (500 MHz, CDCl_3_), δ/ppm: 0.84 (d, 3H, *J* = 7 Hz, C28-CH_3_), 0.86 (s, 3H, C18-CH_3_), 0.90 (s, 3H, C19-CH_3_), 1.00–1.07 (m, 5H, C7-H, C6-H, butyl-3H), 1.15–1.22 (m, 2H, butyl-2H) 1.23 (d, 1H, *J* = 14 Hz, C15-H), 1.26–1.27 (m, 1H, butyl-H), 1.30 (s, 3H, C29-CH_3_), 1.43–1.45 (m, 1H, C1-H), 1.48–1.50 (m, 1H, butyl-1H), 1.50–1.51 ((m, 1H, C9-H), 1.53 (s, 3H, C26-CH_3_), 1.58 (m, 1H, C4-H), 1.61 (s, 3H, C27-CH_3_), 1.68–1.79 (m, 6H, C6-H, C7-H, C2-2H, butyl-2H) 1.83–1.87 (m, 1H, C12-H), 1.93 (s, 3H, C31-CH3), 2.03– 2.12 (m, 5H, C1-H, C5-H, C23-2H, C22-1H), 2.18–2.22 (m,1H, C15-H), 2.23–2.30 (m, 1H, C12-H), 2.40–2.45 (m, 1H, C22-H), 2.90(d, 1H, *J* = 12 Hz, C13-H), 3.42–4.02 (m, m, OC_2_H_4_-24H, C3-H), 4.25 (m, 1H, Hz, C11-H), 5.02 (m, 1H, C24-H), 5.55 (d, 1H, *J* = 8.20 Hz, C16-H).^13^CNMR DEPT-135, 500 MHz, CDCl_3_), δ/ppm = 15.94 (CH_3_), 17.85 (CH_3_), 17.95 (CH_3_), 20.70 (CH_2_), 21.22 (CH_3_), 22.59 (CH_3_), 24.23 (CH_3_), 25.74 (CH_3_), 27.85 (2CH_2_), 29.48 (CH_2_), 29.98 (CH_2_), 30.33 (CH_2_), 32.58 (2CH_2_), 33.33 (CH_2_), 35.63 (CH_2_), 36.08 (CH), 36.30 (CH), 39.31 (CH_2_), 43.13 (CH), 48.21 (CH), 49.18 (CH), 61.75 (2CH_2_), 68.37 (CH), 70.25 (CH_2_), 70.51(CH_2_), 70.53(CH_2_), 70.58(2CH_2_), 70.61(CH_2_), 70.64(CH_2_), 71.38 (CH), 72.65 (CH_2_), 74.098 (CH), 123.40 (CH), ESIMS (*m*/*z*): 817.5 = [M-H], 684.2 = M-3H-COCH_3_ and 538.5 = M-(CH_2_CH_2_)_6_O_7_.

### 3.3. Biological Evaluation 

#### 3.3.1. Antioxidant Assay

Gulcin et al. proposed a method for the free radical scavenging activity (antioxidant activity) using 1,1-diphenyl-2-picryl-hydrazil (DPPH) [68]. An ethanolic solution of DPPH was prepared at a concentration of 0.3 mM. In the next step, different concentrations of each sample were prepared in ethanol, ranging from (62.5–500 μg) by mixing 5 microliters of each sample with 95 µL of DPPH solution. The resulting mixture was transferred to 96-well plates and incubated for 30 min at 37 °C. A microtiter plate reader (Spectramax plus 384 Molecular Device, San Jose, CA, USA) was used to measure the absorbance at 515 nm, and the radical scavenging activity (%) was compared with the methanol-treated control. The standard for the protocol was Butyl hydroxyanisole (BHA). DPPH scavenging effect (%) = Ac − As/Ac × 100, where Ac is the absorbance of the control (DMSO-treated), and As is the absorbance of the sample.

#### 3.3.2. α-Glucosidase Inhibition Assay

The protocol for the α-Glucosidase inhibition assay is built on the formation of product as a result of substrate breakdown, detected by absorbance measurement [69]. In the first step, an enzyme α-glucosidase (Sigma, type III, from yeast) was prepared in buffer solution A (0.1 mol/L potassium phosphate, 3.2 mmol/L-MgCl_2_, pH = 6.8) (0.1 units/mL) mixed with p-nitrophenyl-α-D-glucopyranoside dissolved to obtain the desired concentration of substrate of 6 mmol/L. Another Buffer solution B (102 µL) was prepared by mixing (0.5 M KH_2_PO_4_, 16 mmol/L-MgCl_2_, pH = 6.8) along with 120 µL of sample solution (0.6 mg/mL in dimethyl sulfoxide), 282 µL of water and 200 µL of substrate. In the next step, 200 µL of enzyme was added in the same reaction mixture, incubated for 5 min at 37 °C. After the addition of an enzyme, the mixture was kept at the same temperature for 30 min, and then 1.2 mL, which is 0.4 mol/L, glycine buffer (pH = 10.4) was added to reaction completion. The activity of enzyme inhibition was measured at 410 nm by its absorbance. Acarbose is taken as a positive control. The inhibition% is equal to Ac − As/Ac × 100, where Ac is the absorbance (control), and As is the absorbance (Sample).

### 3.4. Methodology of Molecular Docking

The molecular docking studies were performed to corroborate the antioxidant activity of these compounds. For this study, the three-dimensional crystalline structure of human antioxidant protein, peroxiredoxin 5 (PRDX5), was retrieved from the standard source Protein Data Bank (RCSB: PDB) with PDB ID: 1HD2 at 1.5 Å resolution [70], by using the standard procedure. The synthesized crown compounds **10a**–**d**, along with FABE **8**, were docked into the active site of human peroxiredoxin 5 following the standard procedure using the program Auto Dock Vina 1.2.0 [71].

The 3D structure of human peroxiredoxin 5 with PDB ID: 1HD2 protein was prepared for molecular docking studies using Discovery Studio Visualizer 4.0 (DSV 4.0) [72]. The preparation included the removal of water molecules, the addition of charges and the removal of heteroatoms. The three-dimensional structures of protein and ligands were then saved in PDB and PDBQT format using Open Babel [73]. All the ligands were docked into the active site of the human peroxiredoxin 5. We conducted a blind docking studies, which enabled ligands to independently explore their binding sites and poses without any bias toward the binding pocket. Each docking simulation yielded nine poses. We selected the optimal docked conformation to illustrate molecular interactions. The binding site of 1HD2 protein were identified as Arg A:86, Leu A:96, Lys A:32, Val A:69, Leu A:28, Lys A:63, Val A:70, Gln A:68, Gly A:92, Glu A:16, Ala A:90, Asn A:21, Arg A:95, Gly A:16, Leu A:96. The grid box was set focusing the mentioned binding site at the following parameters: x-dimension = 26, y-dimension = 26, z-dimension = 26, spacing = 0.71 Å, x-center = 7, y-center = 42, z-center = 34, using the program Auto Dock tools [74]. The results of molecular docking studies were expressed in terms of binding affinities in kcal/mol. The results were analyzed using DS-Visualizer software (version 16.1.0.15350). To validate the molecular docking studies of these compounds into human peroxiredoxin 5, a standard antioxidant drug, Butylated hydroxyanisole (BHA), was also docked into the protein using the same parameters. The results consisted of multiple poses for each docked compound. The most appropriate pose with the least binding affinity was selected for further analysis of each ligand.

### 3.5. In Silico ADMET Investigations

The ADMET calculations were performed to predict the potential behavior of the prepared crown ether molecules during pharmacokinetics, i.e., absorption, distribution, metabolism, excretion, and toxicity. These parameters are a significant tool for predicting the pharmacological potential of therapeutic candidates, particularly during the preclinical phase. In this study, Swiss ADME and pkCSM, free and readily accessible web tools (http://www.swissadme.ch/, https://biosig.lab.uq.edu.au/pkcsm/, accessed on 15 August 2024), were utilized to calculate the physicochemical properties, pharmacokinetics, and drug-likeness of the synthesized crown molecules linked with Fusidic acid butyl ester **10a**–**d**. Two-dimensional structures were imported into both websites’ interface in the (SMILES) format, canonical simplified molecular-input line-entry system, and the ADMET properties were generated.

## 4. Conclusions

We successfully prepared novel crown ethers linked with Fusidic acid C-21 butyl ester as potent antioxidants and moderate α-glucosidase inhibitors. All prepared crown ethers were confirmed through their ^1^HNMR, ^13^CNMR DEPT-135, FT-IR, and ESIMS spectral data. Examination of the structure–activity relationship (SAR) showed that the antioxidant potentials of crown molecules increased compared to the Fusidic acid butyl ester **8**, which was used as a precursor for the crown formation. However, the inhibition potential of α-glucosidase decreased. Crown ether (FABE-16-crown-4) **10b** showed highest antioxidant activity with IC_50_ = 22.5 ± 0.2 as compared to butylated hydroxyl anisole used as standard (IC_50_ = 44.2 ± 0.3) as well as showed highest α-glucosidase inhibition (IC_50_ = 23.5 ± 0.2) as compared to acarbose as standard (IC_50_ = 5.2 ± 0.8). Molecular docking investigations of all crown ethers were performed using human antioxidant protein to analyze a binding pattern that showed the minimum number of hydrogen bond interactions and the highest number of alkyl bond interactions for the experimentally most potent antioxidant **10b**. In silico ADMET analyzes were performed for all crown ethers to predict the pharmacokinetic behavior of these compounds. The prediction results showed that these compounds well obeyed (bRO5) and the Veber’s rule for the acceptance as orally administered drugs. All studied ADMET parameters, including Absorption in intestinal mucosa (Caco-2) in cm/s; Human intestinal absorption in (HIA%); Skin permeation (Log Kp); Blood–Brain Barrier partition coefficient (log BB); P-glycoprotein substrate (P-gp substrate); Cytochrome P450 3A4 (CYP3A4 inhibitor); genetic toxicity (AMES toxicity); impairment of Liver function (Hepatotoxicity), were found in acceptable ranges. Overall, present experimental biological activities and in silico ADMET studies showed that the prepared novel FABE-linked crown ethers have promising molecular characteristics for future research to explore biological targets, especially those associated with oxidative stress. Further research is necessary to optimize the bioactivities of FABE-linked crown ethers and to clarify molecular mechanisms.

## Figures and Tables

**Table 1:** In vitro antioxidant and α-glucosidase inhibition activities.

Compound Name	Antioxidant IC_50_ Value (μM)	* Binding Affinities (Kcal/mol)	α-Glucosidase Inhibition IC_50_ Value (μM)
**Intermediate 7**	32.9 ± 0.4	-	75.4 ± 0.7
**8**	54.6 ± 0.2	−6.1	43.2 ± 0.8
**10a**	65.5 ± 0.3	−5.8	67.5 ± 0.8
**10b**	32.1 ± 0.3	−6.7	76.5 ± 0.1
**10c**	22.5 ± 0.2	−6.5	23.5 ± 0.2
**10d**	45.4 ± 0.9	−7.1	54.3 ± 0.1
**Butylated hydroxyanisole**	44.2 ± 0.3	−5.3	-
**Acarbose**	-	-	5.2 ± 0.8

Results are presented as means of triplicate ± standard error of mean (SEM). * In silico binding affinities of favorable interactions with human antioxidant receptor, peroxiredoxin 5 (PRDX5).

**Table 2:** Calculated values of physicochemical parameters.

Compound	MW	LogPo/w	log Sw	HBA	HBD	TPSA	RotB	Vb(RO5)	Vveber
Fusidic Acid **6**	516.71	3.94	−4.294	6	3	104.06	6	0	0
Intermediate **7**	796.60	6.45	−3.721	9	4	126.35	16	0	0
**8**	572.82	5.69	−4.44	6	2	93.06	10	0	0
**10a**	642.91	6.07	−3.79	7	0	80.29	10	0	0
**10b**	686.96	5.96	−3.774	8	0	89.52	10	0	0
**10c**	731.01	6.66	−3.729	9	0	98.75	10	0	0
**10d**	805.09	7.16	−3.573	11	0	117.21	10	0	0

Parameters measured using Swiss ADME, Molecular weight (MW); Octanol/water partition coefficient (LogPo/w); solubility of drug candidate coefficient (Log Sw) where Sw in mol/L; Number of hydrogen bond donors (HBD); Number of hydrogen bond acceptors (HBA); Number of rotatable bonds (RT-b), Topological polar surface area (TPSA); beyond RO5 violation (Vb(RO5)); and Veber violation (Vveber).

**Table 3:** Calculated values of pharmacokinetic parameters.

Compound	CaCO-2 cm/s	HIA (%)	Log Kp	BBB (log BB)	P-gp Substrate	CYP3A4 Inhibitor	AMES Toxicity	Hepato Toxicity
Fusidic Acid **6**	0.632	74.652	−2.735	−0.952	Yes	Yes	No	No
Intermediate **7**	0.796	100	−2.723	−1.393	Yes	No	No	Yes
**8**	0.958	100	−2.543	0.07	Yes	Yes	No	No
**10a**	1.18	99.216	−2.726	−0.736	No	Yes	No	No
**10b**	1.241	99.735	−2.729	−0.921	No	Yes	No	No
**10c**	1.036	100	−2.73	−1.106	No	Yes	No	No
**10d**	1.03	100	−2.733	−1.49	No	Yes	No	No

Parameters measured by using pkCSM; Absorption in intestinal mucosa (Caco-2) in cm/s; Human intestinal absorption in (HIA %); Skin permeation (Log Kp); Blood–Brain Barrier partition coefficient (log BB); P-glycoprotein substrate (P-gp substrate); Cytochrome P450 3A4 (CYP3A4 inhibitor); genetic toxicity (AMES toxicity); impairment of liver function (Hepatotoxicity).

## Data Availability

The dataset is available upon request from the authors.

## Supplementary Materials

The following supporting information can be downloaded at: https://www.mdpi.com/article/10.3390/molecules30092033/s1. [1,30].

## Abbreviations

The following abbreviations are used in this manuscript:

FABE	Fusidic acid butyl ester
BHA	Butyl hydroxyanisole
ADMET	Absorption, Digestion, Metabolism, Excretion, and Toxicity
DPPH	2,2-diphenyl-1-picrylhydrazyl
