# Supplementary material for: Novel Crown Ether-Functionalized Fusidic Acid Butyl Ester: Synthesis, Biological Evaluation, In Silico ADMET, and Molecular Docking Studies
Source: Molecules. 2025 May 2;30(9):2033. doi: 10.3390/molecules30092033 (PMC12073497; doi:10.3390/molecules30092033)
Supplement: Supplementary file 1 [file molecules-30-02033-s001.zip › molecules-3587456-supplementary.pdf]

Supporting Information

Novel Crown Ether-Functionalized Fusidic Acid Butyl Ester: Synthesis, Biological Evaluation, In Silico ADMET, and Molecular Docking Studies

Hira Sultan [1], Nuzhat Arshad [1,*] and Mehreen Lateef [2]

[1] Department of Chemistry, NED University of Engineering and Technology, Karachi 75270, Pakistan; hirasultan@cloud.neduet.edu.pk
[2] Multidisciplinary Lab, Bahria University of Karachi, Karachi 75270, Pakistan; mehreen.lateef80@gmail.com
* Correspondence: nuzhat@neduet.edu.pk

Contents	Page No.
13CNMR spectra	2–6
IR spectra	7–11
1HNMR spectra	12–16
Methods of Computational Investigations	17
Table of Molecular docking studies	18

C-13 Spectra:

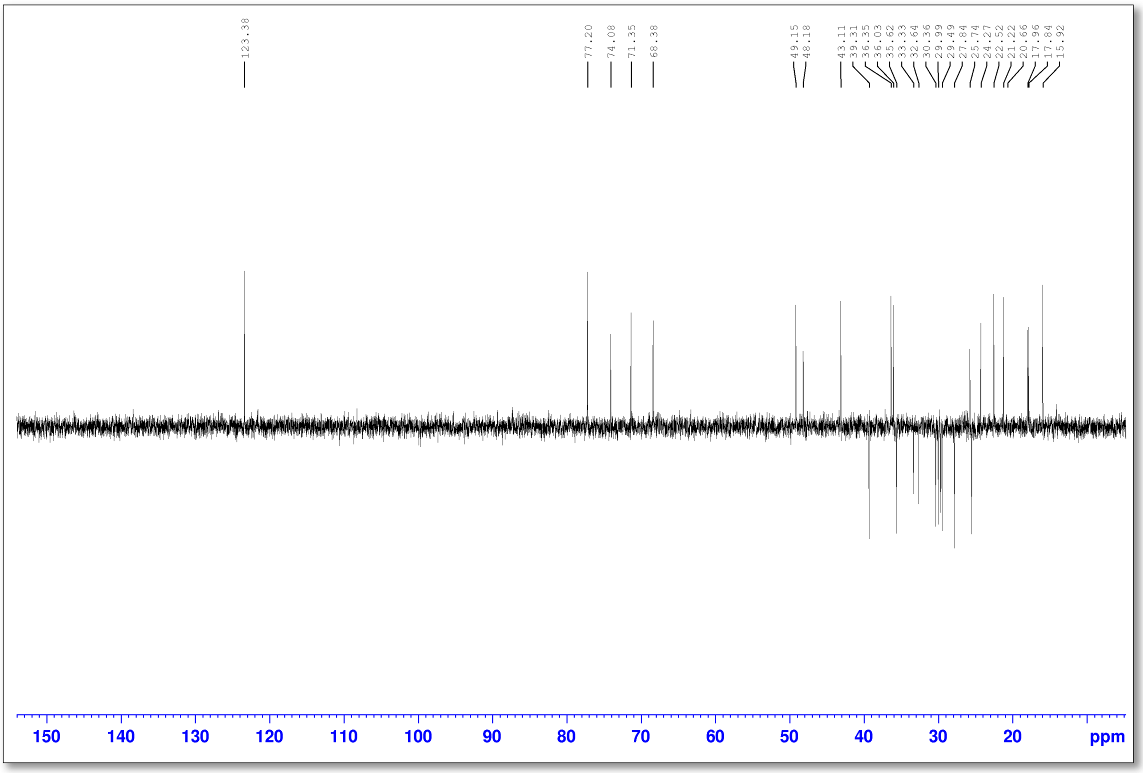

Figure 1. C-13 Dept-135 Spectrum of Compound 8

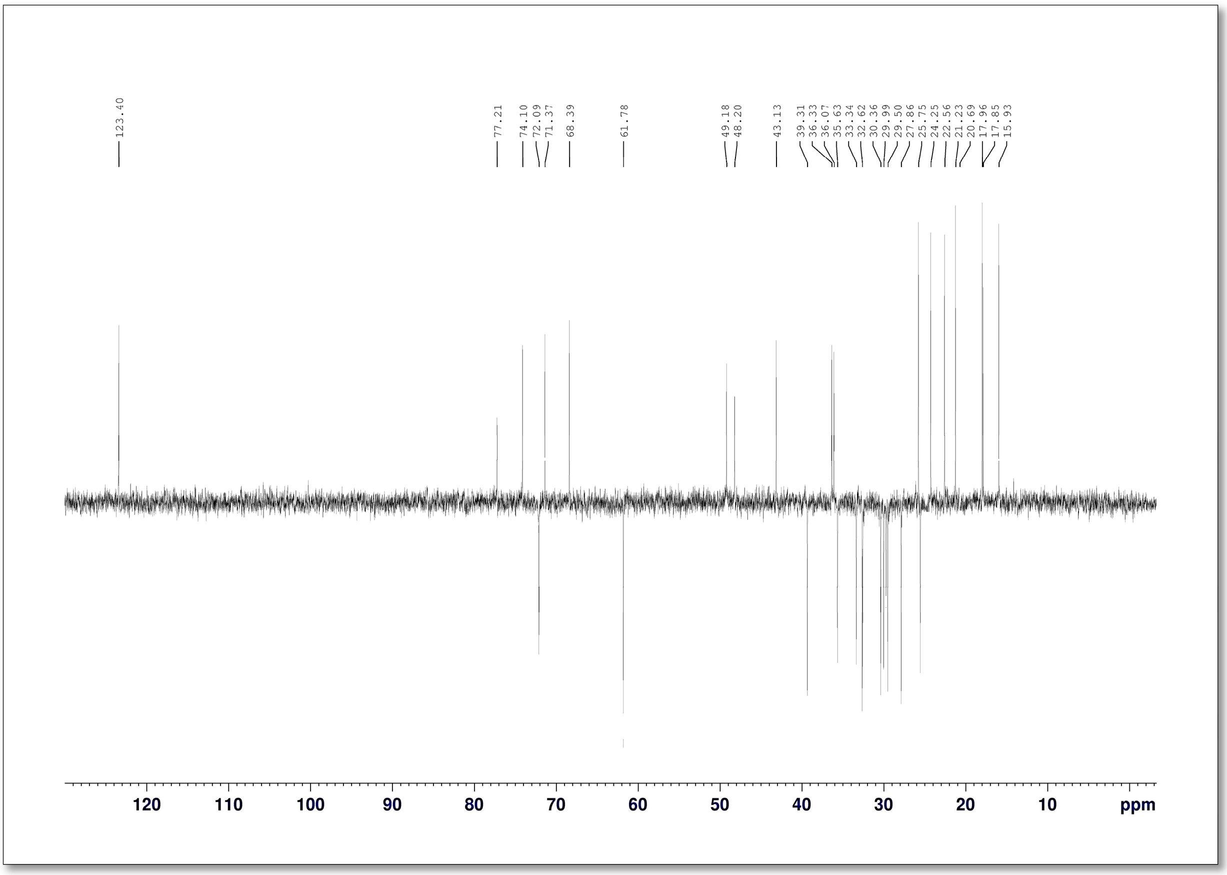

Figure 2. C-13 Dept-135 Spectrum of Compound 10a

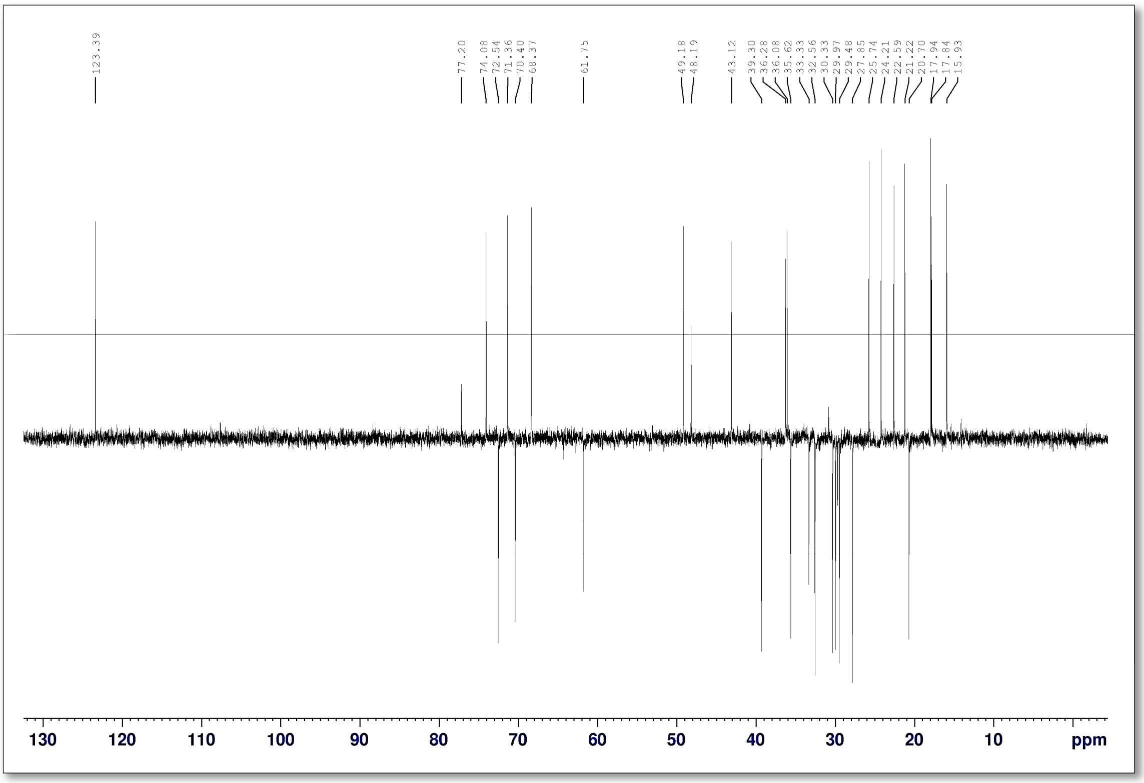

Figure 3. C 13 Dept 135 Spectra of Compound 10 b

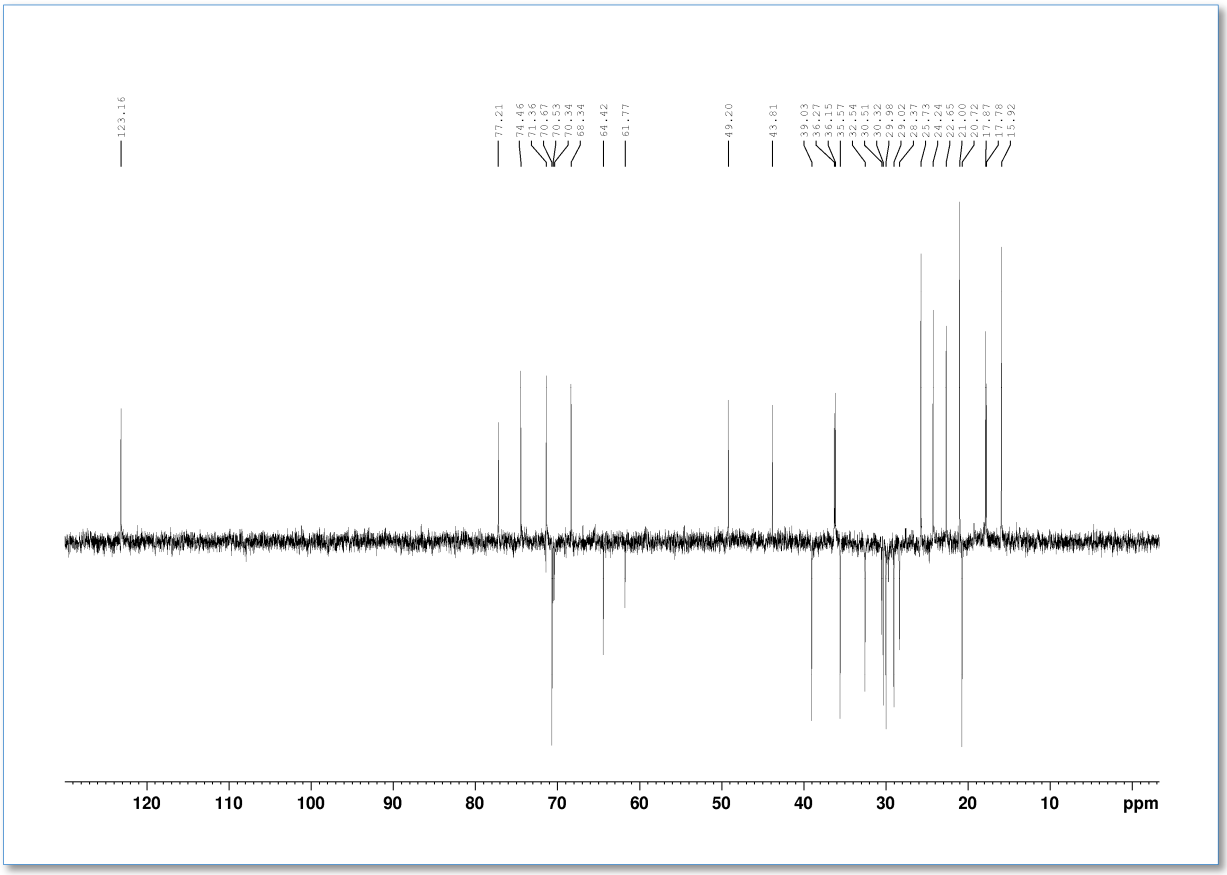

Figure 4. C 13 DEPT 135 Spectra of Compound 10 c

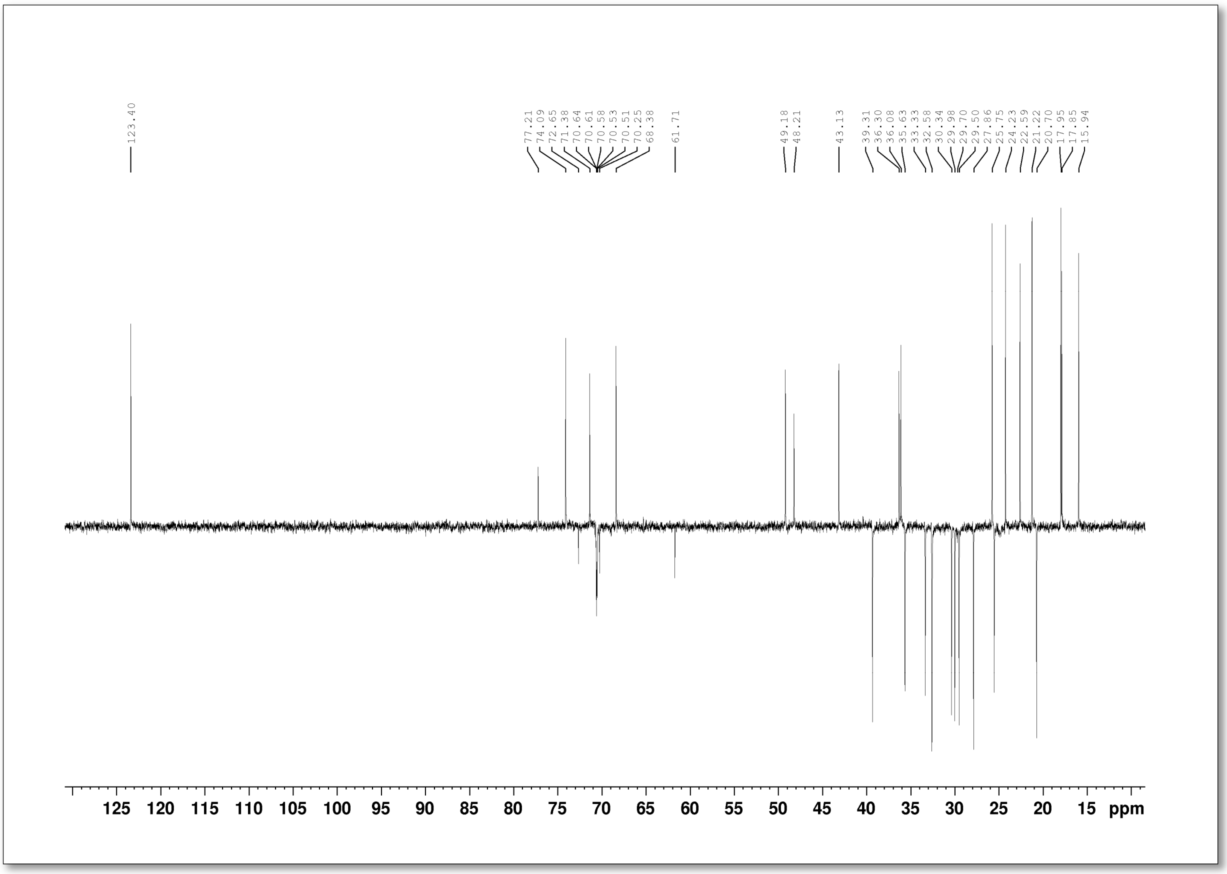

Figure 5. C 13 Dept 135 Spectra of Compound 10 d

IR Spectra

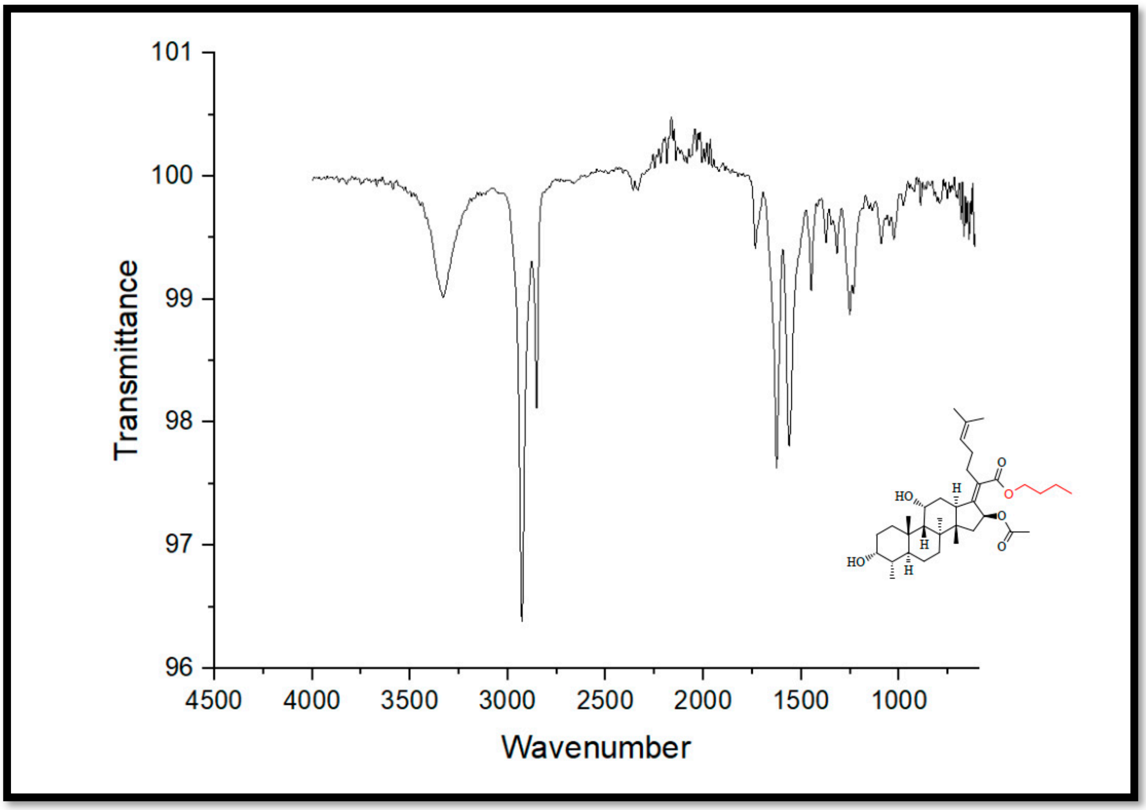

Figure 6. IR Spectra of Compound 8

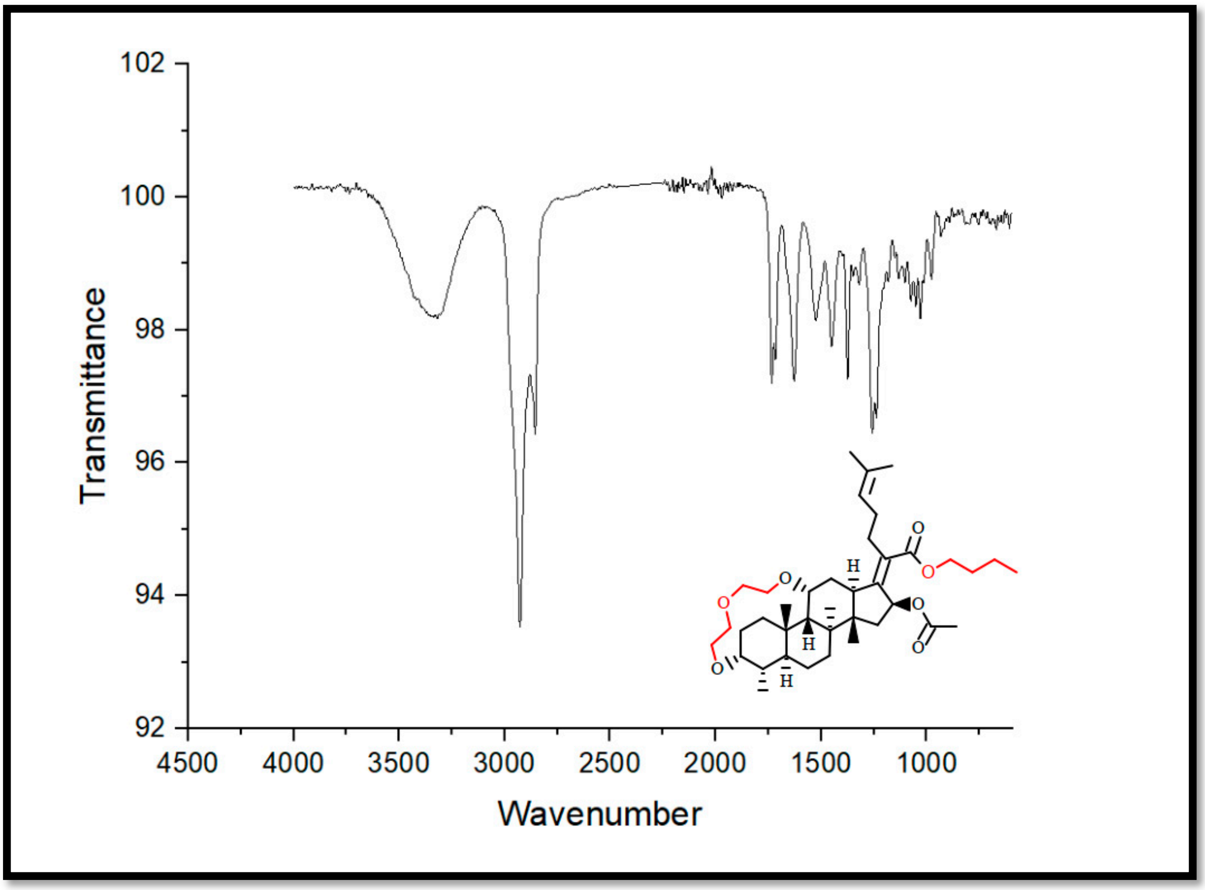

Figure 7. IR Spectra of Compound 10a

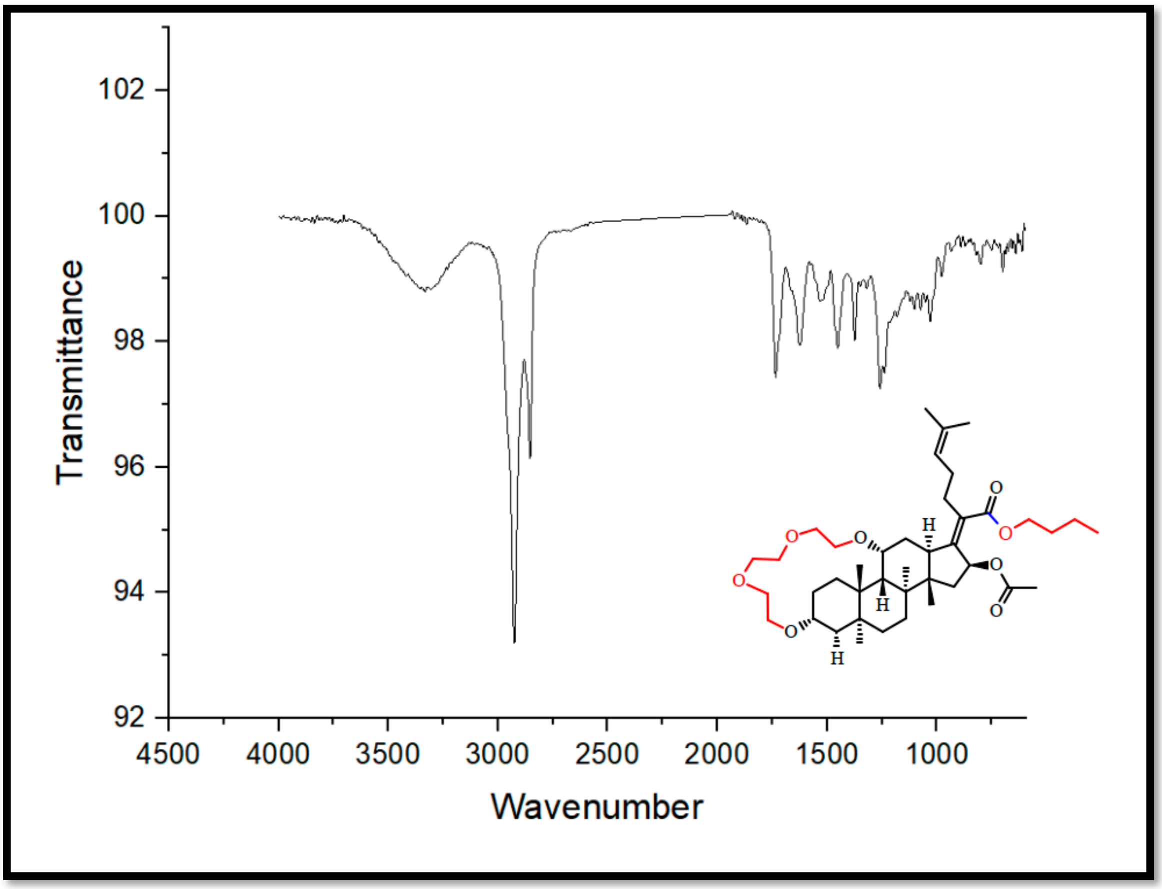

Figure 8. IR Spectra of Compound 10b

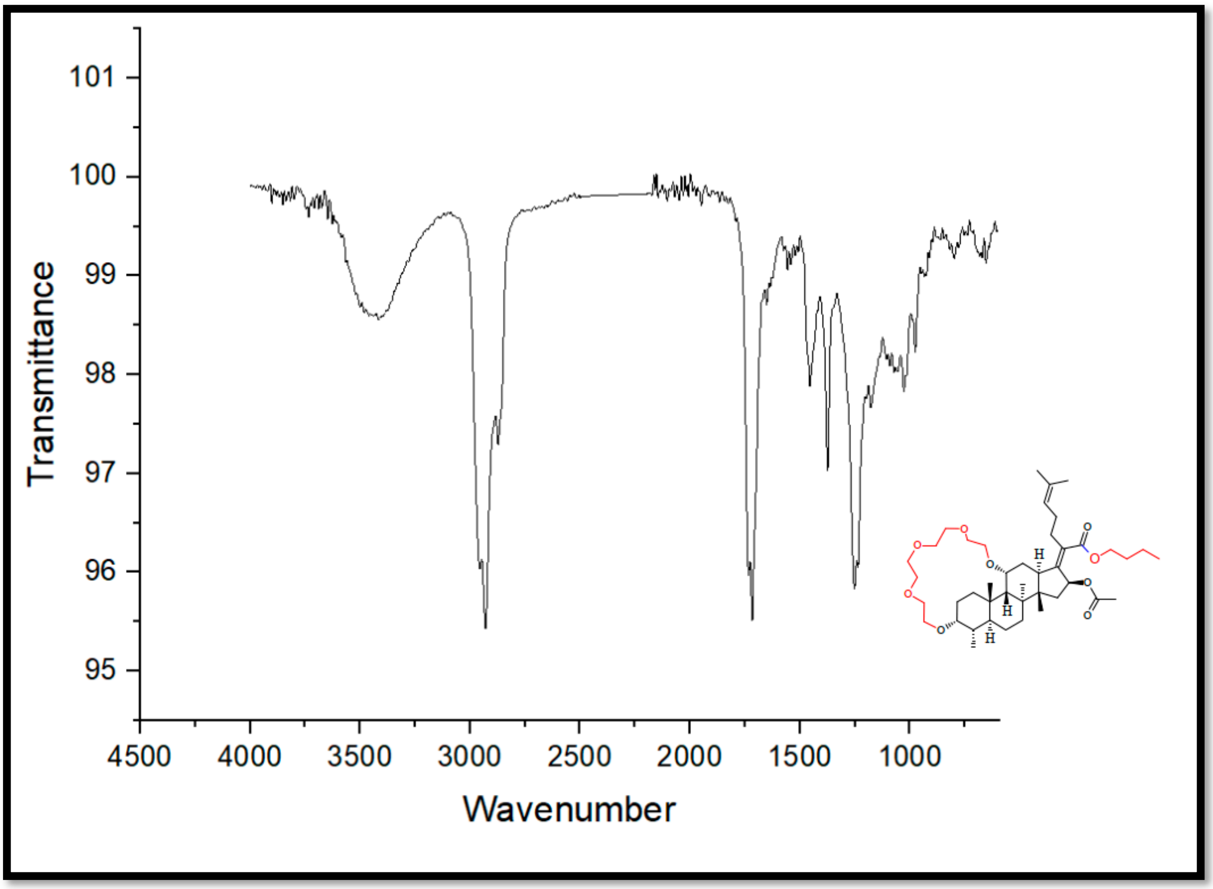

Figure 9. IR Spectra of Compound 10c

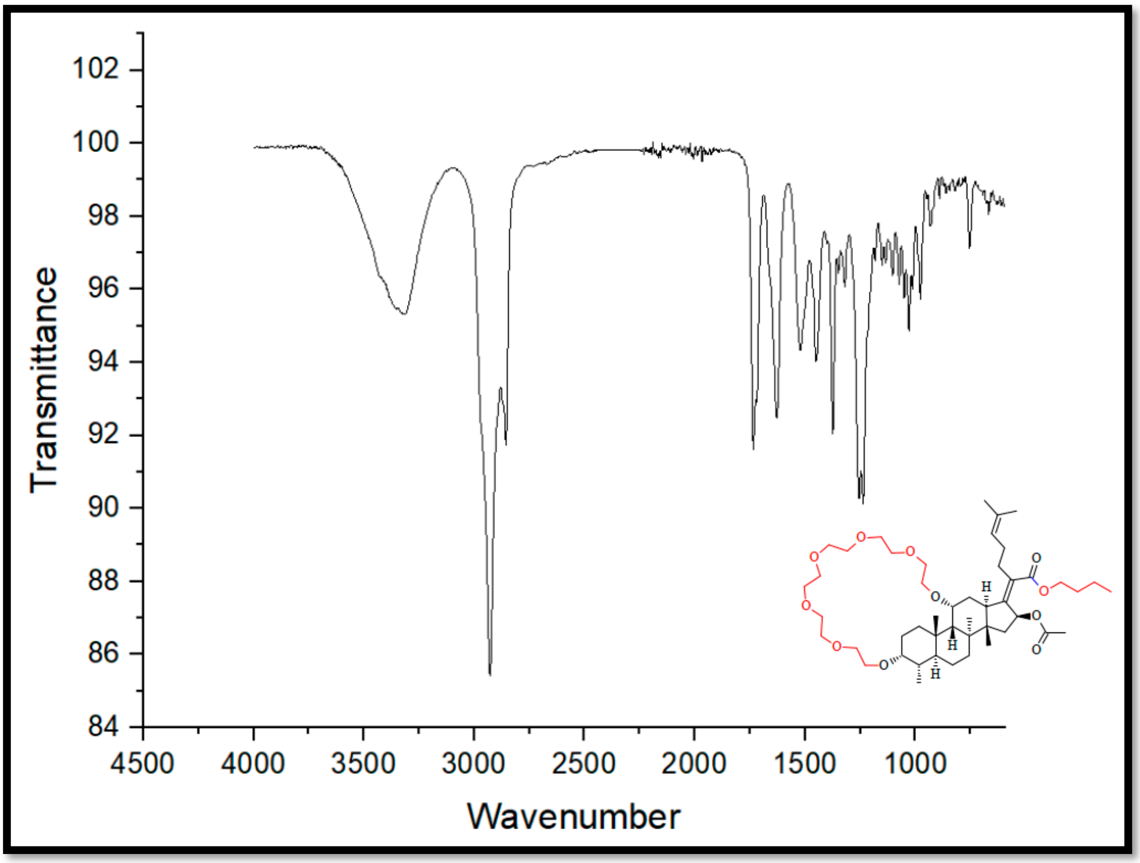

Figure 10. IR Spectra of Compound 10d

H-NMR

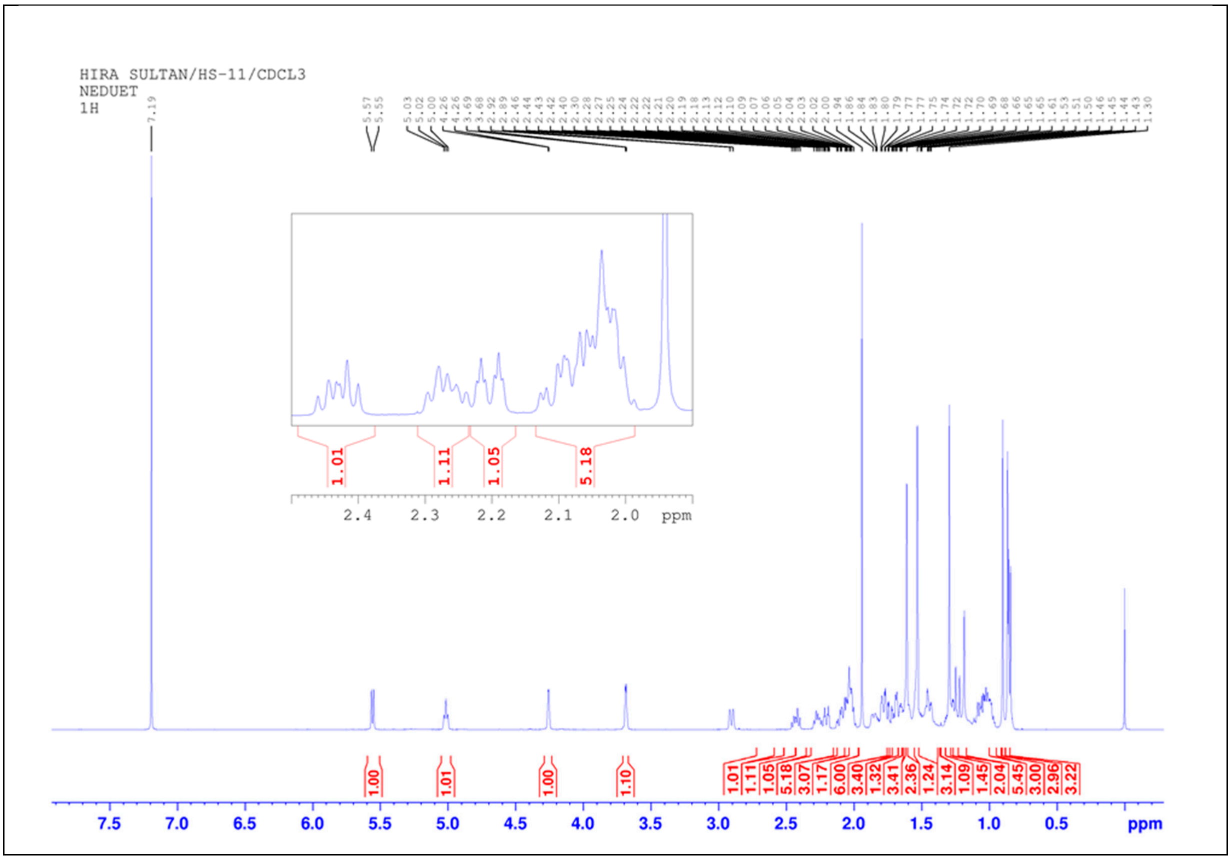

Figure 11. H-NMR Spectra of Compound 8

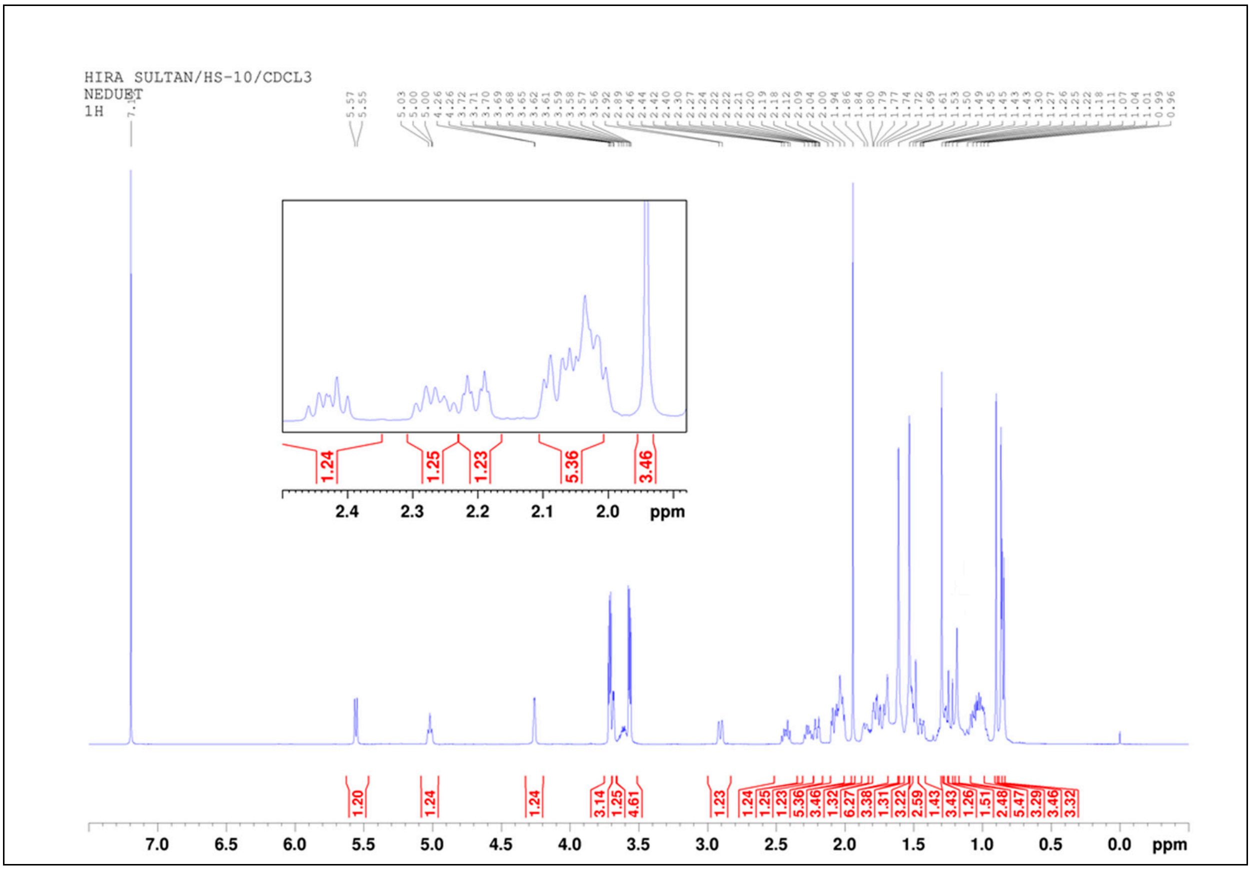

Figure 12. H-NMR Spectra of Compound 10a

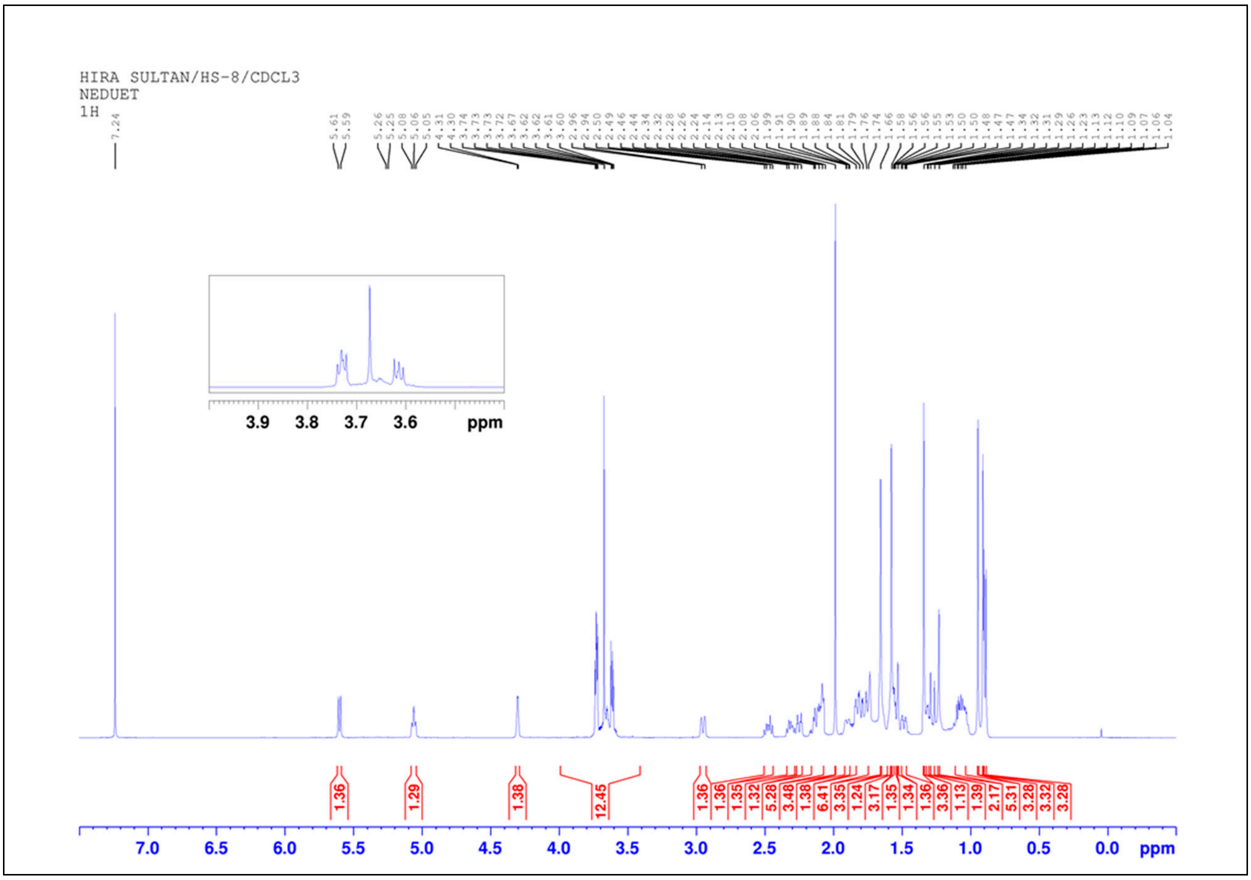

Figure 13. H-NMR Spectra of Compound 10b

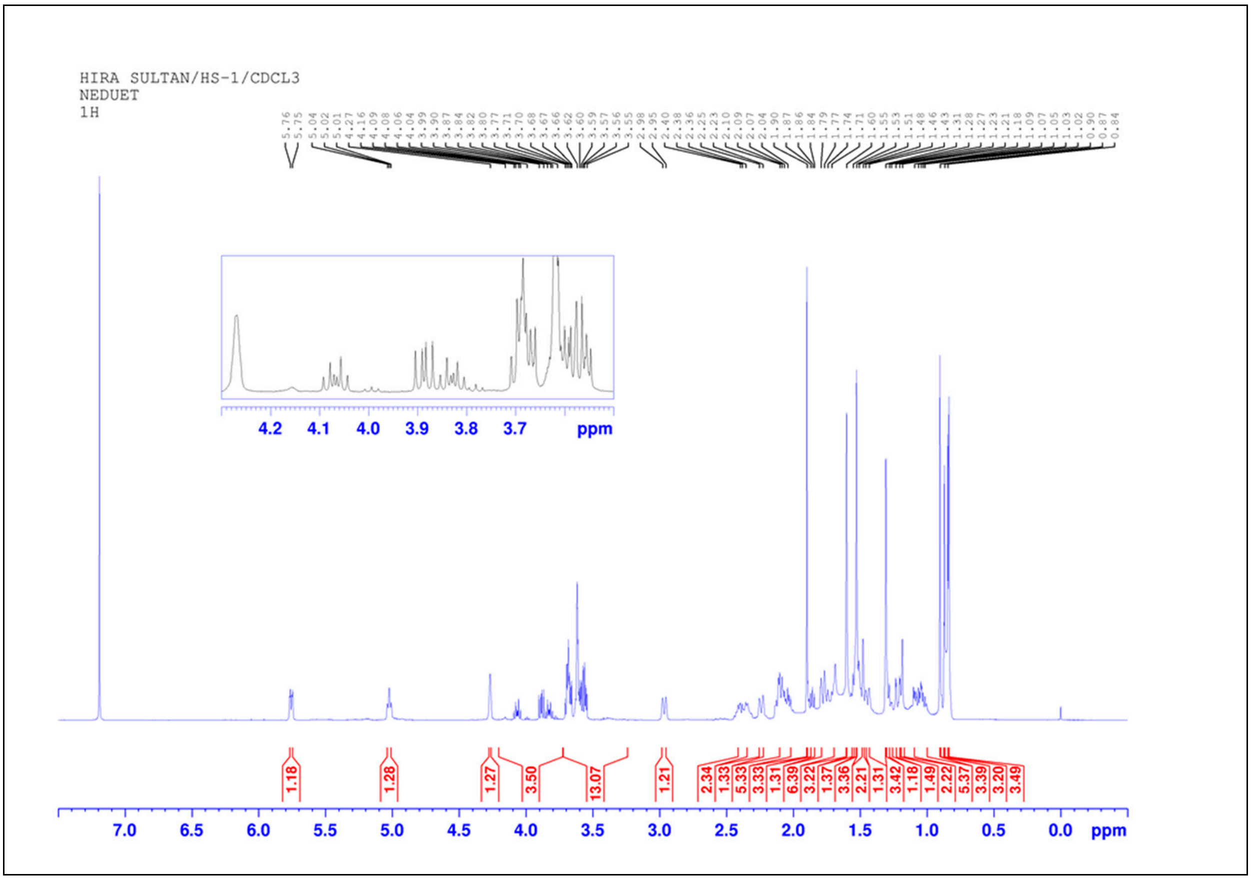

Figure 14. H-NMR Spectra of Compound 10c

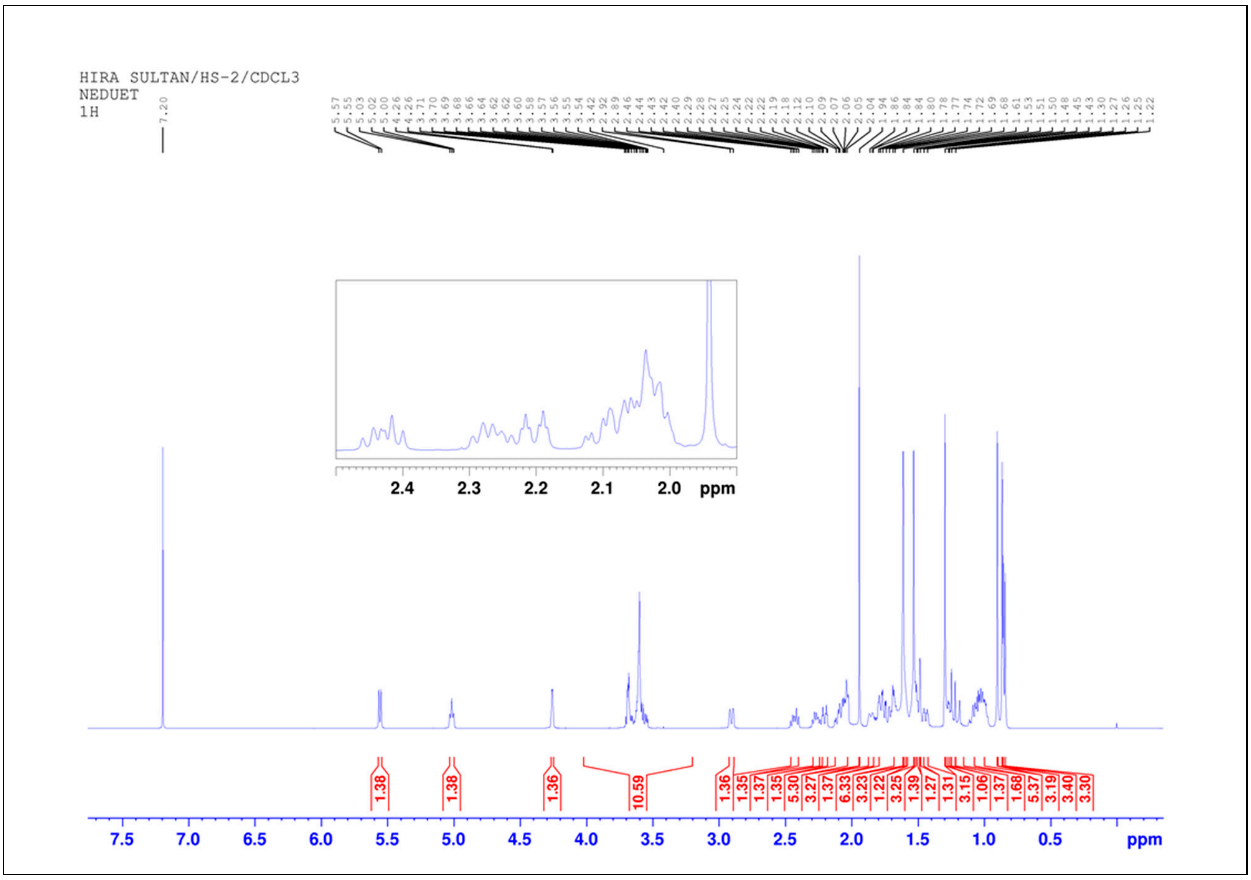

Figure 15. H-NMR Spectra of Compound 10d

Molecular Docking Investigations:

Molecular docking studies were preformed using the standard protocol into the proteins (PDB ID: 1HD2) [1]. Human peroxiredoxin 5 (PRDX5) is a thioredoxin reductase that lowers peroxynitrite, alkyl hydroperoxides, and H_2O_2 (PDB code: 1HD2). A unique kind of mammalian thioredoxin peroxidase called PRDX5 is found cellularly in the cytosol, peroxisomes, and mitochondria. In terms of functionality, PRDX5 has been connected to cellular signal transduction as well as antioxidant protection mechanisms.

For molecular docking, Dr. AutoDock Vina. a free program was used.

The 3D structure of the PDB: 1HD2 protein was obtained from the RCSB (Protein Data Bank) [30], and the necessary preparations were made with the Discover Studio Visualizer 4.0 (DSV 4.0) software, such as cleaning the water atoms in the protein and deleting the other cofactor structure.

The optimizations of **the studied crown** molecule were performed with the Discover Studio Visualizer 4.0 (DSV 4.0) software, recorded in PDB and PDBQT formats with OPEN BEBAL.

Accordingly, the grid parameters of the protein were taken as follows: x_dim=26, y_dim=26, z_dim=26, space=0.71 Å, x_cent=7, y_cent=42, z_cent=34. Therefore molecular docking computations were performed.

Table 2. Docking investigation details, interaction type, and binding score energy (ev)

Molecule	Interacted molecular fragment	Interacted Protein residue	Type of interaction	Binding score
10a	Non polar part C-26	LEU A:116	Alkyl bond	-5.8
	Non polar part ring skeleton	LEU A:149	Alkyl bond	
	Non polar part ring skeleton	LEU A:149	Alkyl bond	
	Non polar part ring skeleton	LEU A:149	Alkyl bond	
	Non polar part ring skeleton	LEU A:149	Alkyl bond	
	Carbon of crown chain	ASP A: 145	Carbon hydrogen bond	
10b	Carbonyl of $–COOC_4H_9$	ARG A:86	Conventional Hydrogen bond	-6.7
	Oxygen of $–COOC_4H_9$	ARG A:86	Conventional Hydrogen bond	
	CH_3 of $–COOC_4H_9$	ARG A:86	Alkyl bond	
	Non polar part C-26	LEU A:96	Alkyl bond	
	Non polar part C-27	LEU A:96	Alkyl bond	
10c	Non polar part C-26	LYS A:32	Alkyl bond	-6.5
	Non polar part C-26	VAL A:69	Alkyl bond	
	Non polar part C-27	LEU A:28	Alkyl bond	
	CH_3 of $–COOC_4H_9$	LYS A:63	Alkyl bond	
	CH_3 of $–COOC_4H_9$	VAL A:69	Alkyl bond	
	CH_3 of $–COOC_4H_9$	VAL A:70	Alkyl bond	
	CH_2 of $–COOC_4H_9$	GLN A:68	Carbon hydrogen bond	
	Oxygen of CH3COO-	GLY A:92	Carbon hydrogen bond	
	Oxygen of crown chain	GLY A:92	Conventional Hydrogen bond	
	Carbon of crown chain	GLU A:16	Carbon hydrogen bond	
10d	Non polar part C-26	ALA A:90	Alkyl bond	-7.1
	Non polar part C-26	LEU A:96	Alkyl bond	
	Non polar part C-27	LEU A:96	Alkyl bond	
	Carbonyl of $–COOC_4H_9$	ARG A:86	Conventional Hydrogen bond	
	Oxygen of $–COOC_4H_9$	ARG A:86	Conventional Hydrogen bond	
	CH_3 of $–COOC_4H_9$	ARG A:86	Alkyl bond	
	Carbonyl of CH3COO-	ASN A:21	Conventional Hydrogen bond	
	Oxygen of crown chain	LYS A:32	Conventional Hydrogen bond	
	Carbon of crown chain	GLY A:92	Carbon hydrogen bond	
8	CH_3 of $–COOC_4H_9$	ALA A:90	Alkyl bond	-6.1
	CH_3 of $–COOC_4H_9$	LEU A:96	Alkyl bond	
	Carbonyl of $–COOC_4H_9$	ARG A:86	Conventional Hydrogen bond	
	Oxygen of $–COOC_4H_9$	ARG A:86	Conventional Hydrogen bond	
	Carbonyl of CH3COO-	ARG A:86	Conventional Hydrogen bond	
	11-OH	GLU A:16	Conventional Hydrogen bond	
	11-OH	ARG A:95	Conventional Hydrogen bond	
BHA	4-OH	GLY A:16	Conventional Hydrogen bond	-5.3
	4-OH	LEU A:96	Conventional Hydrogen bond	
	Π of benzene	ARG A:86	Π Cation	
	Π of benzene	LEU A:96	Π Alkyl	
